# Unveiling the nexus between direct-acting antivirals in hepatitis C virus elimination and immune response

**DOI:** 10.1007/s10238-025-01811-y

**Published:** 2025-07-30

**Authors:** Aya I. Abdelaziz, Eman Abdelsameea, Sara A. Wahdan, Doaa Elsherbiny, Zeinab Zakaria, Samar S. Azab

**Affiliations:** 1 Department of Research and Development, Faculty of Pharmacy, Heliopolis University, Cairo, Egypt; 2 Department of Hepatology and Gastroenterology, National Liver Institute, Menoufia University, Shebin El-Kom, Egypt; 3 Department of Pharmacology and Toxicology, Faculty of Pharmacy, Ain Shams University, Cairo, 11566 Egypt; 4 Faculty of Healthcare Technology, Saxony Egypt University for Applied Science and Technology, Cairo, Egypt

**Keywords:** T cell response, HCV, DAA, Immune checkpoint, CD4+, CD8+

## Abstract

The introduction of direct-acting antiviral (DAA) therapy has been a game-changer in the elimination of hepatitis C virus infection. DAAs treatment achieved higher rates of sustained virological response among HCV-infected individuals across different virus genotypes. DAAs directly target HCV viral several proteins in the HCV lifecycle resulting in controlling the infection. So far, the immune system also plays a crucial role in effective viral eradication. Prolonged antigen exposure, coupled with high viral loads, are key factors that drive immune system failure and the development of chronic infection. T cell exhaustion is the hallmark of the failure of immune response to eliminate the infection. Several sequelae contribute to T cell exhaustion, including the failure of CD8+ and CD4+ T cells, the expansion of the immune suppressive effects of regulatory T cells, and the modulation of epigenetics, which collectively contribute to the persistence of HCV infection. The interplay between DAA therapy and the influence on immune response particularly T cell exhaustion is still an opening question. In this review, we shed light on the recent studies exploring the impact of DAA therapy on CD8+ and CD4+ T cell response as well as the epigenetics change. We also aim to bridge the gap in the new approaches to HCV control.

## Introduction

Hepatitis C virus (HCV) infection has a worldwide distribution, with an estimated 58 million people affected, with 1.5 million new infections every year [1]. Around 30% of HCV-infected individuals clear the virus naturally within 6 months without intervention, whereas most patients develop chronic infection [2]. HCV chronic infection is a major contributor to liver diseases, such as liver fibrosis, cirrhosis and ultimately hepatocellular carcinoma [3]. In the Middle East and North Africa (MENA), viral hepatitis particularly HCV is one of the leading causes of mortality [4].

The host immune response strongly determines the dichotomous outcome of infection (virus clearance versus persistence). The virus’s persistence leading to chronic infection is correlated with several factors including viral immune evasion through viral mutation, suppression of innate immune cells like dendritic cells (DC) and natural killer (NK) cells by HCV viral proteins, and alterations of the innate and adaptive immune response [5]. Despite this, a prolonged adaptive immune response is the hallmark of effective viral eradication. In particular, it has been reported that CD4+ and CD8+ T cell responses have a significant influence on infection outcomes [6]. The introduction of direct-acting antivirals (DAAs) in the management of HCV marked a significant advancement, achieving a high rate of sustained virological responses [7]. However, it is still questioned whether DAA therapies can reverse or restore immune dysfunction specifically T cell exhaustion and the consequences of HCV infection. Therefore, this review will explore the complex relationship between T cells and DAAs in the context of HCV management. Our goal is to identify the gaps in current knowledge and suggest potential areas for future research.

## Review development methodology

We performed a bibliographic search on Medline via PubMed, Scopus, and Web of Science using a combination of specific keywords and MeSH terms, including “Hepatitis C virus,” “Chronic Hepatitis C” “Direct-acting antiviral,” “immune response” and “T cell response”. We encompassed original research articles, systematic reviews, meta-analyses, and clinical trials published in English without restrictions on the time of publication. Synthesis of extracted data will identify trends, patterns, and potential gaps in the literature.

## Overview of HCV infection and cellular immune response

HCV is a single-stranded RNA virus. To date, eight confirmed HCV genotypes (1–8) have been identified [8, 9]. HCV transmission occurs mainly by direct percutaneous exposure to the blood via blood transfusions, unsafe health-care-related parenteral injections, or injecting drug use [10, 11]. Although HCV sexually transmitted is minimal in heterosexual relationships, a recent analysis funded by WHO revealed a high prevalence of HCV infection among men who have sex with men often human immunodeficiency virus (HIV) co-infected [12].

A small number of HCV-infected patients are able naturally to clear the virus during the acute phase. This clearance is associated with rapid response of innate immune genes particularly interferon induced genes, and a slower activation of adaptive immune response. Otherwise, around 70% of patients are unable to clear the virus and instead develop viral persistence.

During HCV infection, monocytes and natural killer (NK) cells, and their interplay with adaptive immunity play crucial roles in shaping the immune response in both acute and chronic phases. Their coordinated actions influence viral clearance, immune regulation, and liver pathology. After HCV entrance and replication into the hepatocytes, HCV alters the expression of several intrahepatic genes, including those involved in the type I interferon (IFN) (IFN α and β), type III IFN (IFN lambda; IFNλ 1- IFNλ4) and several innate immune effector cells, such as NK cells [13]. Several large genome-wide association studies have identified single nucleotide polymorphisms (SNPs) linked to IFNλ3 that are associated with the spontaneous resolution and successful treatment of HCV infection [14–16]. Importantly, HCV has developed several strategies to overcome these responses, for example, by cleavage of important components of the IFN activation cascade by the viral NS3/4a protease or by inactivation of several interferon-stimulated genes [17, 18].

The innate immune response has dual roles as a first line defense against viral invasion and as a contributor to the immunopathology results in liver fibrosis, cirrhosis, and hepatocellular carcinoma. Monocytes and natural killer (NK) cells play pivotal roles in shaping the immune response to HCV [19]. In acute phase, monocytes sense HCV-infected hepatocytes and viral components via Toll-like receptors (TLR), especially TLR3, TLR7, and TLR8. This sensing leads to the production of immunoregulatory molecules such as galectin-9, which can inhibit T cell function and contribute to immune regulation early in infection [20]. Monocytes also secrete cytokines like IL-18 that can activate NK cells, promoting early antiviral responses. HCV directly reprograms monocyte differentiation to promote liver fibrosis and viral persistence [21]. Co-culture of HCV-infected hepatoma cells or cell-free viruses induce monocytes to differentiate into macrophages with mixed M1/M2 polarization profile which in turn increased the production of both pro-inflammatory cytokines (TNF-alpha, IL-1beta) and anti-inflammatory mediators (IL-10, TGF-beta). This polarization has a direct pathological impact on induction the expression of collagen, tissue inhibitor of metalloproteinase 1(TIMP-1), and α-smooth muscle actin (α-SMA) which results in liver fibrosis [22, 23]. Natural killer (NK) cells contribute to the early antiviral defense through cytotoxicity and cytokine production (e.g., IFN- gamma). However, chronic HCV infection often leads to NK cell functional impairment, including altered receptor expression and reduced IFN-gamma production, which contributes to viral persistence and inadequate immune control [24].

The adaptive immune response is mediated by both the humoral and the cellular immune systems. Indeed, regardless of the infection outcome, most HCV-infected individuals develop antibodies against HCV. Some of these antibodies have the ability to neutralize viral particles in a non-strain-specific manner which is crucial in limiting the spread of the virus [25] In the early stages of infection, a rapid and robust induction of neutralizing antibodies is associated with viral clearance, whereas the absence or low levels of antibodies are linked to persistent infection [26, 27]. This makes them valuable for the development of immunoprophylactic products aimed at preventing HCV infection [25].

Overall, although innate and humoral immune responses most likely contribute significantly to the outcome of infection, cellular adaptive immunity has attracted much interest because of its clear impact on infection outcome and potential role in disease progression. Different subtypes of T cells, including CD8+ cytotoxic T lymphocytes (CTLs) differentiate into various subsets, such as central memory T cells, effector memory T cells, and effector T cells. Besides, CD4+ T cells differentiate into different subsets, including T helper (Th)1, Th2, Th9, Th17, Th22, regulatory T cells (Treg), and Follicular Helper T Cells (Tfh). These subsets are distinguished by their unique cytokine profiles and are crucial for the immunological and effector functions of T cells [28]. CD4+ Th1, Th2, and Th17 cells mediate protective immunity against viruses, bacteria, fungi, parasites, and tumors. These effector T cells, however, need to be strictly controlled since they may potentially cause both acute and chronic inflammation, which can result in immunopathology or autoimmunity [29].

Acute HCV infection is characterized by spontaneous HCV clearance due to strenuous and sustained multi-epitope-specific CD4+ and CD8+ T cell responses [30]. The clearance of intracellular HCV is probably mediated by the destruction of virus-infected cells by cytotoxic CD8+ T cells [31]. In addition, data from both in vivo and in vitro models suggests that the process is more sophisticated so that antigen recognition via the CD8+ T cell receptor (TCR) can activate CD8+ T cells to secrete antiviral cytokines (such as IFN γ or Tumor Necrosis Factor-alpha (TNF-α)) leading to inhibition of viral entry, uncoating, and viral replication [31]. Moreover, the development of protective T cell memory may result in spontaneous HCV clearance following reinfection [32, 33]. On the other hand, chronic infection is associated with late, transient, weak, or narrowly focused CD4+ and CD8+ T cell responses [34]. Yet, it is imperative to note that the effects of CD4+ and CD8+ T cell responses are not only vital for viral control but also critical for liver injury and the establishment of liver diseases in viral infections [35]. Evidence drawn from several studies points to the essential functions of both CD4+ and CD8+ T cell responses in viral elimination. It has been reported that changes in liver enzymes and the appearance of clinical symptoms like jaundice are temporally linked to HCV-specific CD4+ and CD8+ T cell responses resulting in a sharp decline in viremia [36, 37]. In the chimpanzee model, which serves as an animal model of HCV persistence, viral clearance is affected by the antibody-mediated reduction of both CD4+ and CD8+ T cells [38, 39]. The failure to completely eradicate HCV infection and the emergence of viral escape mutations in class I major histocompatibility complex-restricted epitopes have been linked to memory CD8+ T cells in adequate control of HCV replication in the absence of sufficient support from CD4+ T cells [38]. Nevertheless, viral replication persisted despite the existence of memory CD4+ T helper cells and did not cease until HCV-specific CD8+ T cells were once again present in the liver [39]. These findings support the notions that memory CD8+ T cells are the primary antiviral effector cells, whereas memory CD4+ T cells assist which is eventually essential for long-term protection from HCV infection. Furthermore, there is substantial immunogenetic evidence for the relevance of different T cells subtypes in HCV clearance. Several studies have reported that certain alleles of the human leukocyte antigen (HLA) class I and II, which regulate CD8+ and CD4+ T cells, respectively, are linked with either the clearance or persistence of HCV infection [40–42]. Therefore, robust responses from CD4+ and CD8+ T cells are crucial for viral clearance.

## Failure of T cell response in chronic HCV infection

A complex crosstalk between CD4+ and CD8+ T cell responses is required for successful viral elimination. The phenotype and functionality of HCV-related CD8+ T lymphocytes are adversely affected by chronic HCV infection, ultimately preventing the virus from being cleared and contributing to liver damage [6].

**Fig. 1:**
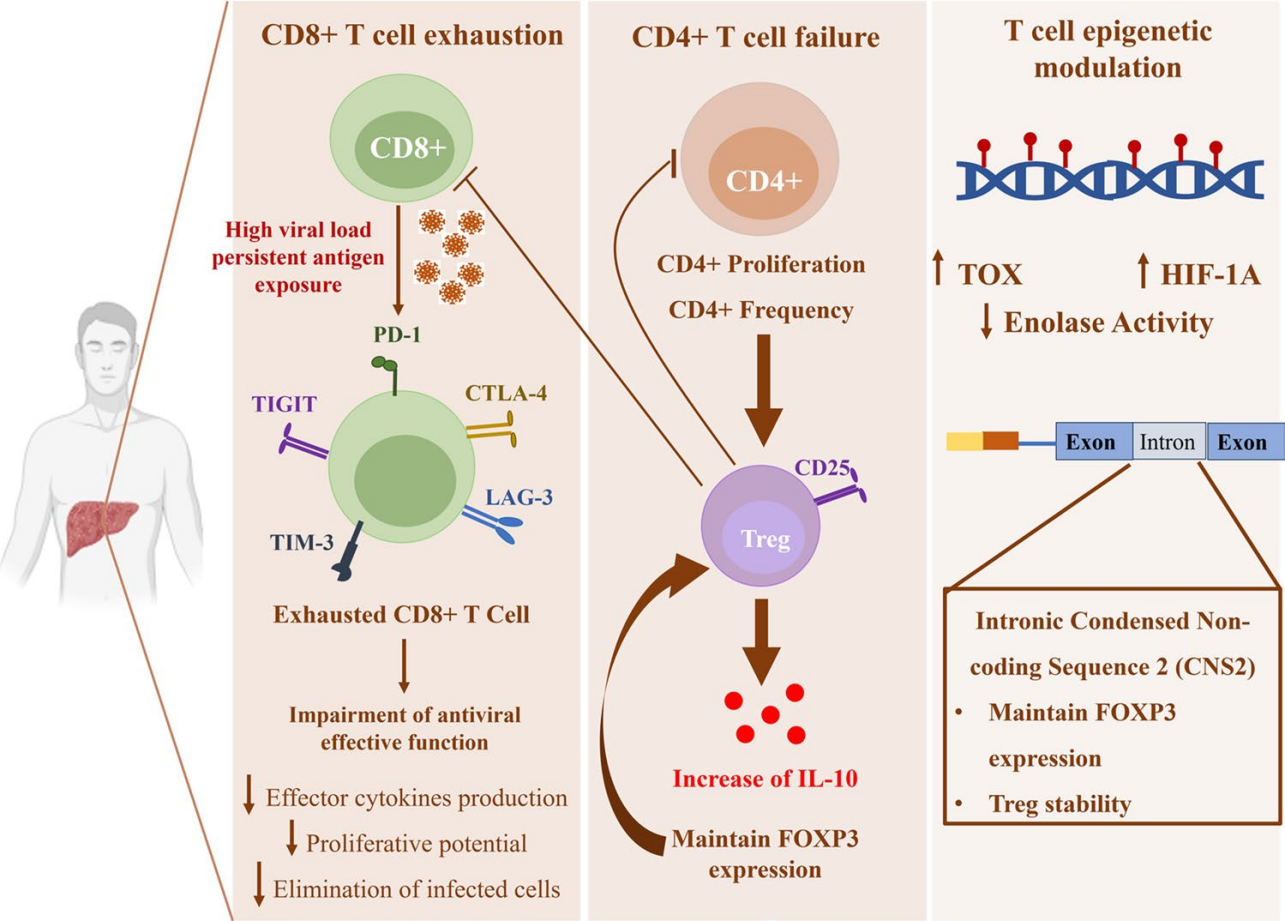
Summary of T cell response modalities in persistent HCV infection. Increased viral load and prolonged exposure to HCV antigens lead to CD8+ T cell exhaustion. Exhausted CD8+ T cells exhibit the expression of immune checkpoint inhibitors such as Programmed cell death protein 1 (PD-1), T cell immunoglobulin and mucin domain-containing protein 3 (TIM-3), Lymphocyte-activation gene 3 (LAG-3), Cytotoxic T-lymphocyte associated protein 4 (CTLA4), and T cell immunoglobulin and ITIM domain (TIGIT). These characteristics impair the antiviral effector function by reducing the production of effector cytokines and the proliferative capacity of CD8+ T cells, leading to failure in viral control. Additionally, CD4+ T cells lose their effector function in supporting CD8+ T cells as they differentiate into a suppressive subset known as T regulatory cells (Treg), which release the inhibitory cytokine IL-10, promoting the production of forkhead box P3 (Foxp3) to sustain Treg cell activation. The persistence of exhaustion results in epigenetic changes in T cells that contribute to the maintenance of exhausted T cells. Exhausted T cells upregulate thymocyte selection-associated HMG BOX (TOX) and hypoxia-inducible factor 1 subunit alpha (HIF1A) while reducing the glycolytic enzyme enolase. Furthermore, intronic conserved non-coding sequence 2 (CNS2) maintains FOXP3 expression, thereby stabilizing Treg cells

As HCV persistence and chronicity are related to T cell functions, various factors are thought to contribute to the lessened T cell responses. Viral mutation and escape can lead to failure of T cells (CD4+ and CD8+ T cells), anergy in CD4+ T cells, exhaustion in CD8+ T cells, activation of regulatory T cells expressing forkhead box P3 (FOXP3+), and impaired dendritic cell function. These are the prevailing causes for diminished T cell responses [43, 44]. The modalities of T cell response leading to persistent infection are shown in Fig. 1.

### *CD8* + *T cell dysfunction in chronic HCV infection*

One of the dysfunctions of T cells during chronic viral infections is T cell exhaustion. This condition, common in chronic infections and cancer, is caused by persistent antigen exposure, resulting in the loss of effector functions, altered metabolism, and distinct transcriptional programs compared to T cells with effector and memory functions [45]. T cell exhaustion is characterized functionally by a decline in cytokines like interleukin-2 (IL-2), reduced proliferative and cytotoxic capacity, impaired pro-inflammatory cytokine production like tumor necrosis factor, and a decrease in the proliferative potential after antigen exposure in vitro [46–48]. It is characterized by increased expression of multiple immune checkpoints like programmed cell death protein 1 (PD-1), CTLA-4, lymphocyte activation gene-3 (LAG-3), and T cell immunoreceptor with Ig and ITIM domains (TIGIT), and T cell immunoglobulin-3 (TIM-3), Fig. 1. At various phases of T cell activation, these checkpoints interact with ligands to restrict activation and suppress activated T cells [49]. The most terminal stages of CD8+ T cell exhaustion correlate with higher viral load and physical deletion of virus-specific T cells [29, 50]. It is the persistent antigen exposure that is believed to be the major factor promoting T cell exhaustion, but CD4+ T cell deletion, activation of regulatory T cells, and increased anti-inflammatory cytokine (e.g. IL-10) production contribute as well [45, 51]. The phenotypic changes, coupled with the immune system’s inability to clear the pathogens, have led to the understanding that persistent infection can exhaust the functional T cell response. This, in turn, impairs the generation of memory CD8+ T cells and predominantly sustains terminally differentiated T cells [52, 53].

Despite their exhaustion, CD8+ T cells are not inert, but, they have modified their strategies to defend the body [45]. Preclinical and clinical studies on HCV infection have identified different subsets of exhausted T cells; cells with preserved ability to proliferate (memory-like CD8+ T cells) and terminally exhausted CD8+ T cells [54, 55]. The hallmarks of memory-like CD8+ T cells are low levels of PD-1, high T-bet expression, transcription factor T cell factor 1 (TCF-1), and IL-7 receptor alpha chain CD127 [56]. Following acute infection, these memory-like CD8+ T cells are partially diverse from actual T_MEM_ cells [55, 57, 58], highlighting the need for more thorough research to better understand the molecular pathways of T_EX_ cells following the elimination of antigens and the obstacles standing in the way of realizing their full memory capability. In contrast, terminally exhausted CD8+ T cells express PD-1 in high levels and increased expression of the T-box transcription factor Eomesodermin (Eomes) resulting in reduced cell turnover capacity [54]. Even while exhausted CD8+ T cells during chronic infections are continuously stimulated by persisting antigens and undergo prolonged massive division, they ultimately fail in pathologic persistent infections [53, 59, 60]. Paley and colleagues explained that persisting antigen stimulation, virus-specific CD8+ T cells utilize two homologous T-box transcription factors to sustain long-lasting antiviral immunity [54]. During a persistent infection, these two cell subsets collaborate to sustain a robust and partially successful CD8+ T cell response, despite their inability to eliminate the virus [54]. Taken all together, it is crucial to maintain the balance between progenitors and progeny in exhausted CD8+ T cells to control antiviral T cell dynamics during chronic infections. Therefore, monitoring these cells over time could help predict the loss of long-term control of chronic infections. Understanding the molecular coordination of this process could lead to strategies for reinvigorating exhausted T cells during chronic infections.

### *CD4* + *T cell responses failure in chronic HCV infection*

CD4+ T cells regulate CD8+ T cell responses and can either support or suppress them depending on the immunological context. In HCV infection, they play a role, in supporting CD8+ T cells by producing cytokines and activating antigen-presenting cells. This support is crucial for initiating and maintaining immunity, which is essential for controlling the infection. Besides, CD4+ T cells have antiviral effects, assist in the maturation of B cells, and regulate immune responses [61]. A robust response from CD4+ T cells is associated with HCV infection outcome. In vivo experiments on chimpanzees showed that CD4+ T cell depletion resulted in persistent, low-level viremia despite functional intra-hepatic memory CD8+ T cell responses [38]. In line with these results, it has been proven that depletion of CD4+ T cells before chronic infection of lymphocytic choriomeningitis virus (LCMV) results in persistent viremia compared to immunological control of the infection in the presence of CD4+ T cells [62]. However, the mechanism underlying the impairment and loss of virus-specific CD4+ T cells in persisting HCV infection remains unclear. In a recent study, Chen and colleagues analyzed the phenotypic and function of HCV-specific CD4+ T cells in acutely infected patients who either spontaneously cleared the virus or progressed to chronic infection. Interestingly, they found no differences in virus-specific CD4+ T cells among the patients during the acute phase. In contrast, in patients who advanced to chronic infection, the frequency of virus-specific CD4+ T cells decreased dramatically [63]. Upon viral control, CD4+ T cells differentiated into memory cells and downregulated inhibitory receptors, while persistent viremia triggered T cell activation and PD-1 and CTLA-4 expression and blocked differentiation, causing the cells to disappear from circulation [63]. These findings are consistent with prior research that found increased inhibitory molecules in the chronic phase of infection [64, 65]. Taken together, these findings suggest that the lack of strong CD4+ T cell responses during chronic infection is due to the rapid loss of the CD4+ T cells themselves rather than a lack of priming. Therefore, a robust CD4+ T cell response is associated with a favorable outcome after infection, highlighting the importance of eliciting this response as an archetype for a successful vaccine.

In the context of persistent HCV infection, CD4+ regulatory T (Treg) cells have garnered attention for their potential role in contributing to weak HCV-specific T cell responses and viral persistence, Fig. 1. Treg cells are one of the key mechanisms regulating self-immunotolerance and homeostasis. They are characterized by the transcription factor FOXP3 and the constitutive cell-surface expression of the interleukin (IL)-2 receptor a chain (CD25) [66]. FOXP3 not only confers cellular identity and functional competence during Treg cell differentiation but also plays a crucial role in their maintenance. Deletion of a conditional FOXP3 allele in differentiated Treg cells leads to a loss of their function [67]. In both mice and humans, FOXP3 mutations and Treg cell dysregulation produce severe autoimmune disease and immunopathology that leads to mortality [68, 69]. Treg cells, particularly the CD4+ CD25+ FOXP3+ subset, are implicated in overregulating HCV-specific T cell responses in chronic HCV infection. Several studies have shown that the abundance of CD4+ CD25+ regulatory T cell activity in chronic HCV individuals may contribute to the persistence of the virus [70, 71]. Furthermore, it is suggested that Treg cells may downregulate responses of CD4+ and CD8+ T cells in persistent infection, particularly within the inflamed liver. The remarkable existence of a dominant prospective Treg population in the livers of chronically infected individuals demonstrates the potential role of Treg cells in maintaining and expanding antigen-specific CD4+ and CD8+ T cells [72]. Additionally, Treg produces IL-10, an immunosuppressive cytokine that helps maintain the expression of the FOXP3 transcription factor, which is necessary for Treg’s suppressive function in other cells [73]. Overall, the regulatory activity of Treg cells in persistent HCV infection may contribute to the weakened HCV-specific T cell responses, thereby facilitating viral persistence.

## Dynamic interactions of FOXP3+ tregs and cytokines: shaping the immune response in HCV infection

There is growing evidence that the dynamic interactions between FOXP3+ Tregs and interleukins (ILs), specifically IL-10, IL-21, IL-6, and IL-17, are important factors in determining the consequences of HCV, ranging from immune-mediated liver pathology to viral persistence. Gaining insight into these immune regulation processes has made it possible to develop treatment plans that attempt to restore equilibrium between pro- and anti-inflammatory pathways.

Key studies have characterized the elevated presence and suppressive functionality of FOXP3+ Tregs in chronic HCV infection, which correlates with impaired effector T cell responses and higher viral loads [74, 75]. Mechanistically, HCV exploits host cytokine networks to promote Treg expansion. For example, HCV-infected hepatocytes secrete TGF-β and IL-10, which favor FOXP3+ Treg induction while suppressing CD4+ and CD8+ effector T cells [76, 77]. Additionally, HCV core protein directly modulates CD4+ T cells, upregulating FOXP3 and IL-10 expression through immune evasion pathways [78, 79]. These findings underscore the role of IL-10 as a central driver of immune suppression and viral persistence, with supporting studies demonstrating that IL-10 blockade enhances effector T cell responses to HCV [70, 80].

Other cytokines, including IL-21, IL-6, and IL-17, mediate nuanced immune effects in HCV infection. IL-21, for instance, enhances effector T cell activity and antagonizes Treg-mediated suppression, promoting viral clearance during acute infection [81, 82]. In contrast, IL-6 destabilizes FOXP3 expression in Tregs while driving inflammatory responses, presenting dual roles in antiviral immunity and immune pathology [81]. Similarly, IL-17, primarily produced by Th17 cells, is implicated in liver inflammation and fibrosis, with elevated IL-17 levels correlating with disease progression [83, 84]. These cytokine dynamics highlight a delicate balance between immune control of HCV and tissue damage.

The Tim-3/Galectin-9 (Gal-9) pathway has emerged as a critical regulator of the Treg-effector T cell balance in HCV infection. Tim-3 expressed on Tregs drives their expansion and immunosuppressive function, while simultaneously inhibiting effector T cells [76, 82, 85, 86]. Blockade of Tim-3/Gal-9 interactions restores antiviral immunity, suggesting potential therapeutic avenues [87, 88].

A potentially underexplored area of research in this context is the role of IL-28, a type III interferon critical for antiviral defense, particularly in hepatocytes. While IL-28 is known to enhance antiviral responses through interferon-stimulated gene activation, its direct interactions with Tregs and FOXP3 expression remain speculative. IL-28 polymorphisms are strongly associated with favorable outcomes in HCV therapy, suggesting a potential role in modulating immune responses, including Treg dynamics [89–91]. In our recent study, we investigated that the impact of SNPs in the promoter region of the FOXP3 gene on FOXP3 expression levels, which in turn influence Treg cell activation in relation to DAA response [92]. Understanding whether and how IL-28 influences FOXP3 and Treg expansion could open new therapeutic avenues, particularly in designing interventions that rebalance immune suppression and antiviral activity.

Collectively, these studies highlight the crucial role of IL-FOXP3 interactions in shaping the immune response to HCV infection, providing valuable directions for therapeutic strategies. Exploring the dynamics between cytokines and Treg_s_ could lead to tailored therapies that target multiple pathways, effectively combating viral persistence and immune-related liver damage. Furthermore, targeting FOXP3+ Treg cells in chronically infected HCV patients may be a promising strategy to enhance antiviral immunity and achieve infection clearance.

## Impact of epigenetic imprints on T cell responses in HCV infection

During HCV infection, epigenetic modifications significantly impact the T cell response. Post-translational histone modifications and DNA methylation are the most prominent epigenetic markers that have been reported to be affected by HCV and to persist [93]. Each T cell subtype has a distinct epigenetic and genetic profile that can be used as a unique marker to identify each subtype. For instance, Treg cells are identified by their high expression of the transcription factor FoxP3. The stability of Treg in response to cytokines is regulated by maintaining FOXP3 levels through demethylation of conserved non-coding sequence 2 (CNS2) in the intronic region [94]. Epigenetic modifications, such as chromatin state, regulate the expression of key effector genes in effector and memory T cells [95]. Compared to naïve T cells, murine memory CD8+ T cells exhibit enhanced effector function during LCMV infection, which is dependent on distinct transcriptional profiles of Ifng, Gzmb, and Prf1 as main effector genes. During the memory phase, there was a decrease in nucleosome density and reduced in interferon-gamma H3K27 methylation and granzyme B chromatin, which persisted. The authors proposed that these chromatin modifications facilitated the rapid up-regulation of effector genes and regulated the memory functions of T cells [96]. Moreover, T cell exhaustion is defined by epigenetics scare that persists across the chronic infection. One of the epigenetic scars associated with critical exhaustion is the presence of super-enhancer elements near transcription factors like TOX and HIF1α. These elements can contribute to a feedback loop that sustains the exhaustion state [95]. Metabolic dysregulation, specifically alteration in mitochondrial functions, is reported in HCV-specific CD8+ T cells during chronic infection [97]. A novel study found that the metabolic regulator Enolase is downregulated in exhausted HCV-specific T cells. The authors propose that boosting the glycolytic enzyme Enolase could potentially restore the effector functions of exhausted T cells [98].

## Clinical outcomes of DAA therapy: unraveling T cell response

Approval of DAAs has rapidly transformed the therapeutic landscape of HCV and provided remarkably high cure rates of over 95% sustained virological response (SVR) [99]. Four classes of DAAs are currently marketed or in clinical development, which are distinguished by their therapeutic target. These classes include nonstructural proteins 3/4A (NS3/4A) protease inhibitors (PIs), NS5B nucleoside polymerase inhibitors (NPIs), NS5B non-nucleoside polymerase inhibitors (NNPIs), and NS5A inhibitors. These drugs vary in their effectiveness against different HCV genotypes and their ability to prevent resistance [100]. Telaprevir and Boceprevir, NS3/4A protease inhibitors, were first approved by the FDA for the treatment of genotype 1 hepatitis C virus [101]. In 2013, sofosbuvir, an NS5B nucleoside polymerase inhibitor was approved for treating genotype 2 and genotype 3 hepatitis C in combination with ribavirin [102]. The combination of Daclatasvir, an NS5A inhibitor, with sofosbuvir, was also approved for treating genotype 1 or genotype 3 hepatitis C [103].

**Fig. 2:**
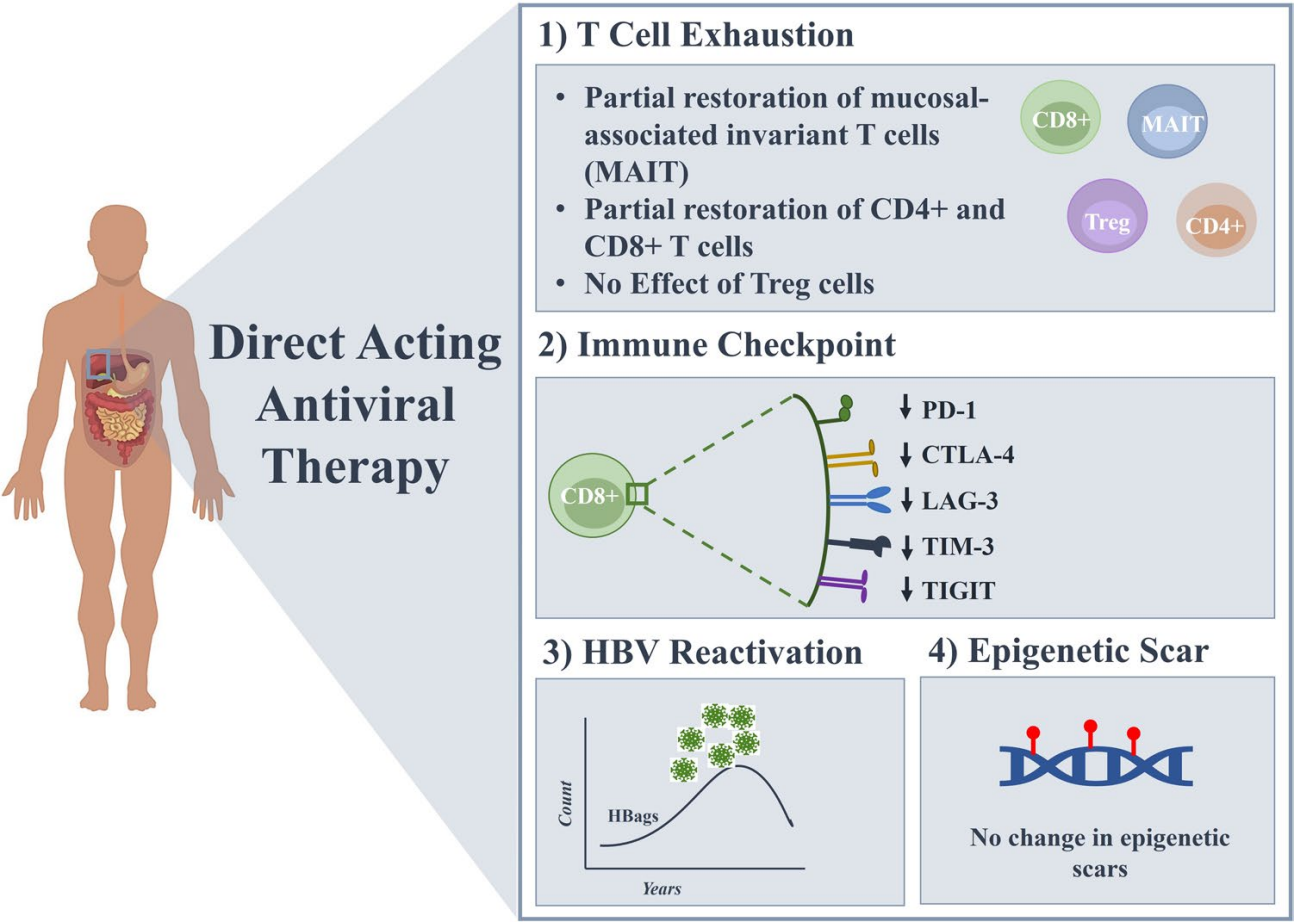
Overview of the immunological consequences of direct-acting antiviral therapy in chronic HCV infection. This schematic illustrates the key immunological effects of DAA therapy in HCV-infected individuals, including the partial restoration of exhausted T cells such as MIAT, CD4+ , and CD8+ T cells, with no observed effect on Tregs. DAA treatment also leads to the downregulation of inhibitory immune checkpoints (PD-1, CTLA-4, LAG-3, TIM-3, TIGIT) on CD8+ T cells. Notably, HBV reactivation may occur in patients with HBV /HCV co-infection following viral clearance. DAA therapy does not impact the epigenetic scars caused by chronic HCV

The development of these agents has also provided researchers with an intriguing novel tool to analyze immune responses to a disease that causes persistent infection for decades before being eliminated from the host within weeks of starting antiviral drugs. While the effect of DAA on HCV elimination is well reported, the impact of DAA therapy on immunological dysfunction remains unclear [104]. Because DAA has a high cure rate, it can inhibit HCV replication and halt chronic antigen stimulation, which might affect the host’s immune system [105]. The notion of immunological scarring, which refers to the long-term impact of persistent HCV infection on the immune system, has gained recognition in recent years [58, 106]. Figure 2 provides an overview of the immunological sequelae during the era of DAA therapy.

Regardless of the course of therapy, DAA-mediated clearance of HCV is followed by rapid downregulation of interferon signaling genes (ISGs) in the blood and liver. HCV clearance is accompanied by hepatic downregulation of type II and III IFNs, their receptors, and ISGs during IFN-free treatment with the DAA, but surprisingly the expression of type I IFN (IFNA2) was augmented at the end of treatment [107]. In addition, type I IFN levels being restored in the liver may help with DAA-mediated HCV eradication and HCV re-infection prevention. This is in line with findings that showed that patients responding to DAA therapy had higher baseline expression of ISGs than individuals experiencing viral breakout [108]. Furthermore, NK cell function returns to normal and their response to IFN alpha is restored after successful DAA treatment [109, 110]. This implies that by impeding viral breakthrough, innate immunity may play a role in clearance of HCV following DAA therapy. Early reductions in viremia and inflammatory cytokine levels are also associated with a decrease in intrahepatic and blood-innate immune cell activation, which is followed by the return of normal NK cell phenotype and function [110]. Nonetheless, the impact of HCV on the diversity of NK cell repertoire persists after viral clearance [111].

The restoration of HCV-induced changes to the immune system, particularly in terms of cytokines and mucosal-associated invariant T (MAIT) cells, has been observed to be not rapidly restored following virus clearance through DAA treatment. MAIT cells are a subset of innate-like T cells predominantly found in barrier tissues like the liver and gut. These cells become activated through exposure to antigens, specifically vitamin B metabolites presented on non-classical MHC molecules, as well as inflammatory cytokines, such as IL12 and IL18 [112]. In HCV-infected livers, the elevation of IL18 derived from monocytes leads to the activation of MAIT cells, consequently causing a decrease in both peripheral and intrahepatic MAIT cell populations [113]. Effective DAA therapy results in a reduction of intrahepatic monocyte activation and a decrease in plasma IL18 levels. This is subsequently followed by a decline in MAIT cell activation and an increase in the frequency of intrahepatic MAIT cells [113]. After the course of therapy, peripheral blood MAIT cell frequency remains diminished for an extended period, spanning many months, and their antigen-dependent effector function does not recover [113, 114]. Additionally, this leads to observable phenotypic alterations and functional impairment [114, 115]. Likewise, several soluble inflammatory mediators do not normalize upon clearance of viral infection [116]. All of these data suggest that long-term HCV infection affects some innate immune system components, even after viral clearance by DAA.

Studies involving T cells’ function during IFN-based therapy for chronic HCV infection indicate a decrease in the quantity and compromised functional capacity of antiviral T cells compared [117, 118] to acute HCV infection, where highly functional T cell responses are observed [118, 119]. This implies that in chronic HCV infection, the endogenous T cell population does not play a substantial role in the success of IFN-based treatment [120]. On the other hand, restoring antiviral immunity, as evidenced by the reversal of exhausted T cell phenotype and immune-driven clearance of residual viral replication, may be necessary for achieving complete remission with DAA therapy. This is because SVR is still achievable even in the presence of HCV-RNA in serum at the end of treatment [121, 122]. In line with this, studies have indicated that peripheral T cell populations are replenished following DAA therapy, with an increase in CD4+ and CD8+ T cells and a shift toward an effector memory population [123–125]. Meissner and colleagues demonstrated a significant increase of CD4+ and CD8+ T-lymphocytes in the peripheral blood early after initiation of the DAA treatment. It is possible that the increased presence of cells expressing CXCR3, a chemokine receptor for CXCL10 that facilitates T cell migration to the liver, is due to hepatic efflux of tissue lymphocytes triggered by changes in inflammation and chemokine receptor signaling following viral antigen clearance [125]. However, a longitudinal case–control study found higher CD4+ and CD8+ T cell activation levels in HCV and HCV/HIV-1 patients, but no significant influence of DAA therapy on activation status [126]. In line with this, it has also been shown that chronic HCV infection induces an expansion of regulatory CD4+ T cells that also appears to persist long after DAA-mediated HCV clearance [127]. Thus, the immunosuppressive persistence of Tregs may contribute to complications of liver disease even long-term after HCV cure. However, Vranjkovic and colleagues revealed a link between the severity of liver fibrosis in chronic HCV infection and persistent dysfunction of CD8+ T cells, even after successful viral clearance with DAA therapy [128]. Individuals with advanced liver fibrosis (F4) exhibit persistent alterations in CD8+ T cell subsets and increased cytotoxic activity, which does not improve after DAA treatment. This study highlighted that the prolonged hyperactivity of CD8+ T cells following DAA treatment may carry substantial long-term consequences including compromised antitumor immunity, a risk of aggressive forms of hepatocellular carcinoma (HCC), increased chances of HCC recurrence [129], and a greater susceptibility to extrahepatic cancers [130]. Additionally, concerns extend to the potential difficulty in generating effective responses to an HCV vaccine and an increased vulnerability to HCV re-infection [131].

While DAA therapy restores the functional capacity of circulating CD8+ T cells specific to HCV, particularly their proliferation, it does not significantly affect the number of these cells in circulation [132]. The restoration of their proliferative capacity is linked to alterations in the composition of the HCV-specific CD8+ T cell population. Terminally exhausted CD8+ T cells diminish after the removal of the antigen, whereas memory-like CD8+ T cells exhibit memory-like traits such as antigen-independent survival and recall proliferation [55]. Furthermore, in a patient experiencing a virologic relapse after DAA therapy, HCV-specific CD8+ T cells displayed recall responses and antigen re-exposure in patients who relapsed after DAA therapy. However, they failed to mediate viral clearance, resulting in the development of an exhausted terminal phenotype [55]. Another recent study revealed imprint in virus-specific CD8+ T cells in cirrhotic chronic genotype 1 hepatitis C patients treated with DAA [133]. It is noteworthy that essential transcriptional regulators continued to be in the exhausted state, and functionally, the exhausted T cells did not exhibit much improvement. As a result of mutations in the virus, T cells in HCV chronic infection were exposed to antigens for shorter durations, but they exhibited greater functional and transcriptional similarities to memory T cells from spontaneously resolved HCV infection. The duration of T cell stimulation therefore affects the recovery from exhaustion, because antigen removal following prolonged exhaustion is insufficient for the establishment of functional T cell memory [134]. These findings is consistent with a study on chimpanzees, which showed that HCV-specific CD8+ T cells persisted following DAA-mediated HCV elimination but did not provide protection against reinfection [135]. Furthermore, dynamic changes in ex vivo HCV-specific CD8+ T cells after viral clearance are reported and showed suppression in patients experiencing poor viral outcomes [136].

Collectively, it is suggested that DAA therapy may partially restore different T cell components and functions in exhausted CD8+ T cells. However, complete recovery is not achieved, indicating that persistent antigen exposure leaves lasting effects on the T cell repertoire. This has implications for reinfection and vaccination after DAA treatment for chronic HCV infection.

Compared to CD8+ T cells, there have been few studies on the changes in the CD4+ HCV-specific T cell subset induced by DAA treatment. One to two weeks after the start of the therapy, an increase in T cells expressing the CXCR3 chemokine receptor in the peripheral blood was detected [125]. This suggests an early emigration of liver-infiltrating lymphocytes to the blood, as CXCR3 is expressed on the majority of liver-infiltrating CD4+ T cells in chronic infection [137]. In line with these observations, serum levels of the IFN-inducible cytokine IP-10 (protein product of the CXCL10 gene, the ligand for CXCR3) are rapidly downregulated after DAA initiation [107]. It has been demonstrated that HCV-specific CD4+ T cells exhibit an exhausted phenotype during chronic infection [64], and it remains to be investigated if the expression of inhibitory receptors persists after the virus is eliminated and if memory formation can occur. Smits et.al. identified a pool of HCV-specific CD4+ T cells characterized by a follicular T helper cell signature, which remains present even after the persistent infection is eliminated by DAA therapy [138]. These cells appear to play a role in preserving HCV-specific humoral immunity since their presence in the circulation is correlated with a decrease in both HCV-specific neutralizing antibody titers and germinal center activity. In contrast, single-cell transcriptomic analyses of T cells in persistent HCV-infected patients did not observe changes in the frequency of these CD4+ T cell groups in the overall T cell population [139].

CD4+ /CD8+ ratio is a molecular marker associated with markers of T cell activation and innate immune activation particularly for non-AIDS-related events [140]. The post-HCV elimination CD4+ /CD8+ ratio following DAA-based therapy remains poorly understood, with conflicting findings in the available results. A recent study on CHC patients revealed a slight decrease in CD4+ /CD8+ ratio at the end of DAA treatment which is to be found a good predictor of HCC [141]. Conversely, Rossotti and colleagues demonstrated no notable alteration in the CD4+ /CD8+ ratio during a 12-week follow-up of DAA therapy [142]. Future research is needed to explore the determining impact of the CD4+ /CD8+ ratio after HCV cure.

As mentioned earlier, Tregs gradually expand and accumulate in the liver. They suppress antiviral T cell responses, which determine the progression of liver disease after HCV clearance [6]. Therefore, it is important to determine whether Tregs are normalized after HCV is cured with DAA therapy. Langhans and colleagues found that the frequencies and activation status of FOXP3+ CD25+ CD4+ T cells remained elevated compared to normal controls in both DAA treatment groups, even persisting long-term after HCV elimination [127]. In line with these observations, the frequency of Tregs did not show significant changes after DAA treatment. Moreover, Treg cells increased in patients with hepatitis C cirrhosis and correlated with the fibrosis stage [143]. An additional study revealed a gradual decline in both the frequency and inhibitory function of Tregs from the baseline to the end of treatment, followed by an increase from the end of treatment to SVR 12 in chronic hepatitis C patients undergoing DAA therapy [144].

Thus, the prolonged presence of immunosuppressive Tregs may contribute to complications of liver disease even in the long-term after HCV cure potentially including, high susceptibility to re-infection, lack of protective immunity after DAA-induced HCV clearance, or possibly a persisting increased risk for liver cancer.

## DAA-mediated modulation of immune checkpoints in viral infections

As discussed earlier, chronic HCV infection leads to CD8+ T cell exhaustion, which reduces the effector function of T cells. It is hypothesized that this exhaustion is a protective mechanism against the tissue-damaging consequences of a prolonged immune response caused by persistent infection [45]. Exhausted T cells are identified by the presence of immune checkpoint (IC) inhibitory molecules, such as programmed death (PD-1), cytotoxic T-lymphocyte-associated antigen 4 (CTLA-4), T cell immunoglobulin and ITIM domain (TIGIT) and T cell immunoglobulin-3 (TIM-3). The interaction between these molecules and their ligands induces intracellular signaling that inhibits cytokine production and limits T cell activation [145]. Additional factors, including insufficient support from CD4+ T cells, immunosuppressive cytokines, and signals provided directly by inhibitory receptors also contribute to the development of T cell exhaustion [146, 147]. Notably, blocking the PD1/PDL1 inhibitory pathway can restore the function of exhausted virus-specific CD8+ T cells [148–150].

In this context, the immunomodulatory role of DAA is still under investigation. In Table 1, we highlight the existing clinical research that have examined the effects of effective HCV eradication by DAA therapy on immunological checkpoint pathways. Nevertheless, several unanticipated side effects have been reported, indicating a potential impact of DAAs on the host immune response. These include increased occurrences of herpesvirus or hepatitis B virus (HBV) reactivations, a higher prevalence of autoimmune diseases either developing spontaneously or being triggered, and contradictory results regarding the elevated rates of early recurrence of HCC [151–153]. After DAA therapy, broad phenotypic changes in immune T cells were reported such as the expression of inhibitory molecules PD-1 and CTLA-4 [123, 134]. On the other hand, a recent study has shown that DAA-induced HCV elimination is vital in inhibiting PD-1 and CTLA-4 and their ligands, which are known to play key roles in suppressing T cell activation against malignancies [154].

**Table 1:** Studies reporting the effect of DAA therapy on immune checkpoint in patients with HCV infection

Reference	Model	Immune checkpoint	Results
Osegueda et al. [163]	Peripheral blood mononuclear cells (PBMC) were isolated from patients coinfected with HIV/HCV and collected before, at the end of, and 12 months after successful DAA treatment	PD-1, TIGIT, and TIM-3 expression	Only PD-1 was differentially expressed, with a higher percentage of PD-1 + cells in individuals with advanced fibrosis
Shive et al. [164]	HCV patients initiated direct-acting anti-viral therapy (n = 15) and were followed longitudinally	PD-1 and TIGIT	There was a reduction in the proportions of co-expression of CD4 + T cell TIGIT + CD57 + and CD8 + T cell PD-1 + CD57 + after DAA therapy in HCV patients
Bi et al. [165]	Proteomic study on CD56 + NK cells of chronic HCV-infected patients before and 1 year after DAA therapy (sofosbuvir and ledipasvir)	TIM-3	A sustained lower surface expression of TIM-3 was reported although the total amount of cellular TIM-3 analyzed by proteomics was found widely normalized 1 year after therapy
Li et al. [154]	47 chronic HCV-infected patients who received asunaprevir and daclatasvir were collected before and 24 weeks after treatment	PD-1, PD-L1, CTLA-4, 4-1BB, OX40	- The frequency of T cells expressing PD-1, PD-L1, CTLA-4, 4-1BB, OX40 were decreased - After viral, the frequency of PD-1-expressing T cells declined in most phenotypes, including CD4 + , memory CD4 + , and CD8 + - In the non-responder group, no significant decrease in the frequency of these cells expressing PD-1 was noted
Llorens-Revul [106]	27 chronic HCV-infected patients undergoing DAA treatment	PD-1, TIM-3, LAG-3	There was a marginal decrease of PD-1, TIM-3, and LAG-3 expression on CD4 + T cells but these differences did not reach statistical significance
Ramadan [166]	Cohorts of Chronic HCV patients, HCV-related cirrhosis without HCC, HCV-related cirrhosis, and HCC treated/untreated with DAAs	CD8/PD-1 and CD19/PDL-1	After DAAs therapy, exhausted CD8 + T cells (expressing PD-1), and B cells expressing PD-L1 were significantly decreased
Szereday et al. [167]	Patients were treated for 12 weeks, including dasabuvir, ombitasvir, and paritaprevir/ritonavir plus ribavirin combination treatment	PD-1, TIM-3 and their ligands PD-L1, Gal-9	DAA treatment decreased the expression of inhibitory checkpoint receptors and their ligands such as Tim-3 by immature/regulatory NK bright and NKT-like cells, PD-L1 by NK cells, and Gal-9 by NK cells and monocytes
Romani et al. [162]	Cohorts of HCV patients treated for 4 weeks with DAA therapy [ledipasvir/sofosbuvir / Vedroprevir and ledipasvir/sofosbuvir / Vedroprevir/ Radalbuvir] regimens for 4 weeks who either achieved sustained virologic response (SVR)	PD-1, Tim-3, CTLA-4 and 2B4	Frequencies of the PD-1 expressing subsets (PD-1 + Tim-3 + and PD-1 + CD160 +) were significantly higher at baseline in patients who achieved sustained virological response (SVR) than in relapse group and at the end of treatment: PD- 1^hi^, PD-1 + 2B4 + PD- 1 + Blimp1 + , PD 1 + KLRG1 + were increased in SVR group CD4 + T cells with higher expression of PD-1 + CD160 + , PD- 1 + Blimp-1 + in SVR than relapsers Higher frequencies of Eomes + PD-1 + CD8 + cells and lower frequencies of CTLA-4 expressing PD-1 − CD4 + T cells in SVR relative to relapse at baseline
Shrivastava et al. [123]	Patients coinfected with HIV/HCV who were successfully treated with combination DAA treatment regimens of daclatasvir and asunaprevir for 24 weeks, daclatasvir/asunaprevir /beclabuvir for 12 weeks, and sofosbuvir and ledipasvir for 12 weeks	PD1, 2B4, and TIGIT	The changes in the expression of PD1 in CD4+ T cells and CD8 + T cells and TIGIT in CD4+ T cells and CD8+ T cells decreased with daclatasvir/asunaprevir /beclabuvir for 12 weeks of treatment
Tonnerre [134]	20 long-term HCV-infected patients who received 12 weeks of paritaprevir/ritonavir/ombitasvir + dasabuvir + ribavirin	PD-1, TIGIT, and TIM-3	The transcriptional data show that escaped T cells (T_ESC_) do not exhibit changes in key pathways such as PD-1 response signaling or in their memory phenotype after HCV cure, in contrast to T_EX_ after cure, the expression level PD-1 was similar to those of T_MEM_ most HCV-specific CD8 T_EX_ cells continued to express T cell inhibitory molecules, most T cell inhibitory molecules were significantly decreased in either percentage (2B4, CD39, TIM-3, and CTLA-4) or median fluorescence intensity (MFI) (PD-1, CD95, and TIGIT)

Programmed cell death protein 1 (PD-1), Programmed cell death protein 1 ligand (PD-L1), T cell immunoglobulin and mucin domain-containing protein 3 (TIM-3), and Lymphocyte-activation gene 3 (LAG-3), T cell immunoreceptor with Ig and ITIM domains (TIGIT), Galectin-9 (Gal-9), Exhausted T cells (T_EX_)

After successful therapy by DAAs, a subset of virus-specific T cells persists long-term but does not significantly restore effector function [55, 58]. Nonetheless, the mechanisms responsible for these persistent functional deficits and whether the cessation of chronic stimulation can reset the exhausted state are still unclear. Phenotypic changes in exhausted T cell populations that remained clonally stable after the viral cure suggested a transition toward a memory-like state. However, there was limited functional enhancement in these cells, and key transcriptional regulators remained in an exhausted state [134]. The investigation of the influence of HCV treatment modality on T cells remains a subject of inquiry. In a recent study, non-cirrhotic HCV patients treated for the first time with DAAs and achieved viral elimination showed a significant decrease in PD-1 expression levels compared to cirrhotic patients treated previously with IFN-α [106]. The difference in liver damage and the more weakened HCV-specific immune response may be related to the finding that T cells derived from cirrhotic liver display a more profound state of functional exhaustion. Moreover, the persistence of T cell exhaustion following DAA treatment in subjects previously treated with IFN may be attributed to the phenotypic shifts in T cells induced by IFN. In addition, reversing T cell exhaustion is less likely in older patients which may contribute to HCC development in advanced age [106]. A study suggests that IL-7 plus 4-1BBL treatment; an immune checkpoint TNF receptor superfamily member 9 (4-1BB)/4-1BB ligand (4-1BBL) which improves the HCV-specific CD8+ T cell response; could improve HCV-specific cytotoxic T cell reactivity in patients with short/mid duration infections [134, 155]. On the other hand, the duration of the disease is a critical factor in determining the extent of T cell exhaustion. It has been reported that T cells that undergo shorter TCR stimulation as a result of viral mutation have the capacity to transform into memory-like cells that retain their functionality [134]. Consequently, early intervention to reinvigorate exhausted T cells by targeting exhaustion mediators can rescue more terminally exhausted T cells. Additionally, patients with long-term illnesses often experience more severe T cell exhaustion, necessitating additional PD-1/PD-L1 checkpoint blockade to enhance T cell proliferation capacity [156].

Researchers are trying to find novel ways of restoring T cell responses in patients with chronic HCV infection. A recent study examined the critical function of TNF receptor-associated factor 1 (TRAF1) signaling, suggesting that immunotherapy targeting this pathway may be a potential treatment approach for patients with malignancies and persistent viral infections. TRAF1 serves as a positive regulator of T cell activation, and its suppression by pathogens is utilized as a strategy to evade targeted adaptive immune responses [156, 157]. In LCMV infection, the expression of TRAF1 is upregulated by interleukin-7 (IL-7), whereas TGF-β1 downregulates it [158]. Thus, upregulating TRAF1 induced by IL-7 could be a beneficial strategy for cases where exhausted peripheral effector memory cells are still detectable [156].

PD-1 plays a role in regulating T cell responses during HCV infection, and the ex vivo blockade of PD-1 blockade has been successful in reactivating HCV-specific T cells [159]. However, it is unclear whether PD-1 blockade can reverse T cell exhaustion in infected patients. Studies show that only one in three infected chimpanzees respond to anti-PD-1 treatment [160], and only 11% of HCV-infected patients experience significant suppression of viral replication [161]. Pre-existing virus-specific T cells in the liver are believed to be essential for enhancing T cell responses. In contrast, Romani and colleagues (2019) surprisingly revealed that PD-1 T cells expressed multiple inhibitory molecules and they were increased in the responder group treated for 4 weeks of DAAs therapy than relapsers [162].

Thus, the impact of DAA therapy on immunity remains controversial, suggesting that a potentially more effective approach could involve the combination of immune checkpoint inhibitors with a therapeutic vaccine (Box 1).

**Box 1:** Immune responses in HCV infection

Dual role of innate immunity	Monocytes and NK cells act as a first defense line against HCV but also promote liver inflammation and fibrogenesis
NK cell dysfunction	Chronic HCV induces altered receptor expression and functional exhaustion of NK cells promoting viral persistence
Humoral responses and neutralization	Rapid, robust antibody induction correlates with spontaneous clearance, highlighting their potential for prophylactic vaccine
Significance of T-cell immunity	Sustained multi-epitopes CD4 + and CD8 + T-cell responses are critical for eradication

## HBV/HCV co-infection: implication of HBV reactivation in DAA therapy era

While DAAs are not considered immunosuppressive agents, there is a potential for HBV reactivation in individuals with co-existing HBV and HCV infections undergoing DAA therapy. It has been reported that coinfection of HBV and HCV creates a unique situation where the reciprocal relationship between HBV and HCV viral kinetics often occurs. In this scenario, hepatitis B surface antigen (HBsAg) remains detectable alongside low-level or even undetectable HBV viremia, while high-level HCV viremia is present [168]. Another possible explanation is that HCV triggered the innate immune response, which then suppressed HBV replication [169]. Although DAAs achieved SVR in HCV-infected patients, a report from the U.S. Food and Drug Administration (FDA) has reported 29 cases of HBV reactivation in patients with HBV-HCV co-infection treated with DAAs, two of which resulted in death and one required liver transplantation [170]. Another meta-analysis on HBV reactivation in HBV-HCV co-infected patients found that the estimated rate of HBV reactivation in studies using interferon-free DAA-based therapy (12.2%) was not significantly different from that in studies using interferon-based therapy (14.5%) [171]. Additionally, HBV reactivation occurs earlier with DAAs (4–12 weeks during treatment) compared to interferon-based therapies (typically at the end of treatment or later). Studies on DAA-based therapies were more likely to document hepatitis resulting from HBV reactivation (12.2% with DAAs vs. 0% with interferon) [171]. In agreement with this meta-analysis, two recent studies approved the risk of HBV reactivation with DAA therapy in HBV/HCV coinfected patients [172, 173]. These observations can be explained as follows: First, DAA treatment leads to a rapid and significant decrease in HCV viral load, which results in an early reduction of viral interference mediated by the host’s immune responses as observed in patients with HCV and HBV coinfection [174, 175]. Secondly, unlike DAAs, interferon also suppresses HBV replication and can result in a sustained HBV response in a significant number of patients after 48 weeks of treatment [176]. On the other hand, another meta-analysis reported HBV reactivation in 24% of HBsAg-positive patients, while only 1.4% of patients with resolved HBV infection experienced reactivation after DAA treatment. In addition, there was no HBV-related hepatitis reported in patients with a resolved infection while 9% of patients with chronic HBV infection had the risk of HBV-reactivation-related hepatitis [177]. Nevertheless, the FDA released a black box stating that before initiating treatment, all patients receiving DAA therapy for HCV infection should undergo testing for HBV to check for past or current HBV infection [178]. Moreover, the current guidelines of the American Association for the Study of Liver Diseases and the Infectious Diseases Society of America (AASLD) [179] and the European Association for the Study of the Liver (EASL) [180] been updated to include testing for current HBV infection or previous HBV exposure before initiating HCV treatment. Given the current lack of understanding regarding the molecular mechanisms underlying HBV reactivation during or after DAA therapy, further research is warranted in this domain.

## Impact of DAA therapy on B cell dysregulation

Chronic HCV infection is strongly associated with B cell dysregulation, manifesting as benign lymphoproliferative disorders like mixed cryoglobulinemia (MC) and malignant B cell non-Hodgkin’s lymphomas (B-NHL) [181]. HCV infects and multiplies in B cells through the HCV envelope protein E2, which interact with CD81 expressed on B cells [182]. Memory B cells are the primary HCV infection reservoirs among B cell subsets, mostly due to their extended lifespans [183]. Persistent HCV infection in B cells can lead to lymphoproliferative disorders, potentially progressing to B cell lymphomagenesis (MC) [182]. A recent study reported found that there was no significant improvement in mixed cryoglobulinemia vasculitis in HCV patients treated with DAA therapy [184]. Therefore, the presence of HCV in peripheral B cells can make it more difficult to treat CHC patients with antiviral drugs.

## Emerging of resistance-associated substitutions (RAS) development under DAA therapy

Resistance-associated substitutions (RASs) emerge under DAA pressure in HCV, representing a significant challenge to treatment efficacy. RASs result from amino acid mutations in DAA-targeted nonstructural (NS) proteins—NS3/4A protease, NS5A protein, and NS5B polymerase—that disrupt inhibitor binding while preserving viral replication fitness [185, 186]. Development and persistence of RASs are influenced by structural changes, fitness costs, and compensatory mutations, as well as host immune dynamics.

**Fig. 3:**
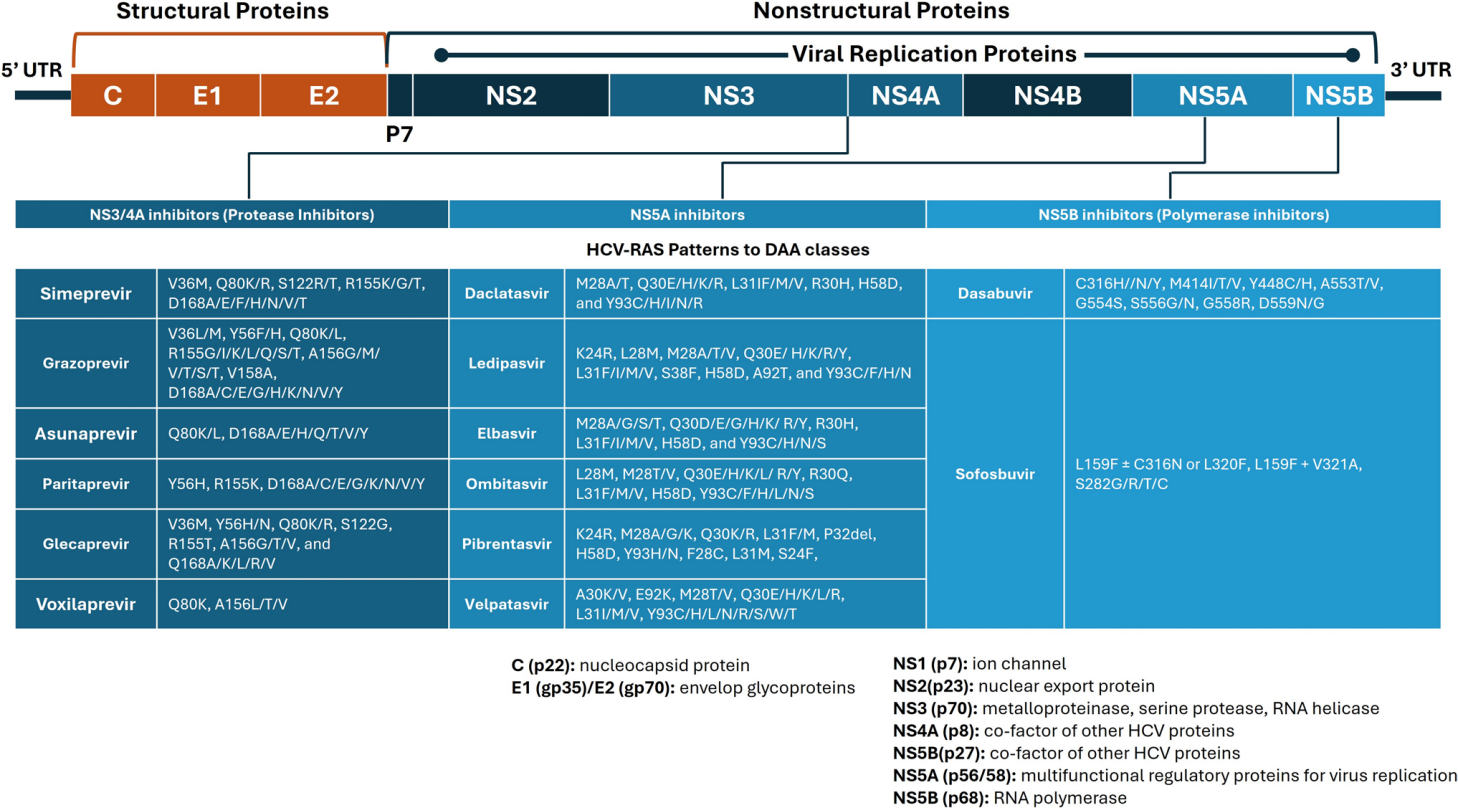
Hepatitis C virus genome organization, direct-acting antiviral targets, and key resistance-associated substitutions (RASs). The hepatitis C virus (HCV) genomic structure is shown schematically, with the locations of viral proteins and the targets of direct-acting antivirals (DAAs) highlighted. NS3/4A protease inhibitors, NS5A replication complex inhibitors, and NS5B polymerase inhibitors are the three main groups of DAAs shown in the figure. Clinically relevant resistance associated substitutions (RASs), which show genetic variants connected to treatment resistance, are listed beneath each DAA class and are associated with particular antiviral medications illustrating genetic changes connected to treatment resistance

Key RAS hotspots include NS3 residues A156, R155, D168, NS5A-Y93H, and NS5B-S282T, which are associated with resistance to protease inhibitors, NS5A inhibitors, and nucleotide analogues, respectively [185–187]. Structural studies reveal that RASs reduce inhibitor binding affinity through direct steric hindrance, allosteric effects, or conformational instability, while computational analyses (e.g., molecular dynamics simulations) show disruptions in energy and interaction networks critical for drug-target binding [188–190]. Although many RASs incur fitness trade-offs, compensatory mutations in the HCV genome restore replication efficiency, particularly when RASs occur in mutational networks or quasispecies dominated by viral diversity [186, 191]. Genotype-specific variation is evident, with GT3 being uniquely resistant to DAAs due to structural instability in NS3/4A protease, frequent NS5A RASs (e.g., Y93H), and poor virologic response [192, 193]. Figure 3 summarizes the common reported RSA against DAA.

As we discussed earlier, chronic HCV infection is marked by immune exhaustion, with functional impairments in CD4+ helper T cells and CTLs. Exhausted CD8+ T cells downregulate effector functions and fail to effectively target HCV-infected hepatocytes, particularly when RASs overlap with CTL epitopes, enabling dual DAA resistance and immune escape [194]. Similarly, weakened CD4+ T cell responses impair CD8+ T cell priming and antibody formation, allowing viral persistence and expanding the quasispecies reservoir for RAS emergence [195]. Furthermore, Tregs further suppress antiviral immunity, promoting immune tolerance of resistant variants, especially in genotypes prone to multi-DAA resistance [189]. Additionally, suboptimal immune responses or immune exhaustion (e.g., high PD-1 expression on CD8+ T cells) allow fitness-compensated resistant variants to dominate under DAAs, contributing to treatment relapse [186, 196].

The emergence of RASs under DAA therapy reflects a complex interplay between adaptive mutations in the HCV genome and host immune dynamics. Future treatment strategies should integrate genotype-specific resistance profiling with immune-modulating approaches to limit RAS selection and improve patient outcomes.

## Epigenetic remodeling in DAA therapy

Multiple virus, host, and environmental factors play a multifactorial role in the development of HCV-induced HCC. HCV creates a carcinogenic environment in the liver through persistent inflammation, production of viral proteins, disrupted cellular signaling processes, and oxidative stress, leading to genetic and epigenetic instability in the host [197]. This might be explained, for example, by the fact that HCV infection can have a significant impact on the epigenome and that many of these alterations persist as “scars” in various cell types, such as hepatocytes and CD8+ T cells, even after the virus is eradicated [95, 198]. There is still an opening question on how epigenetic mechanisms influence T cell exhaustion and the possibility of reversing the exhausted cell state after successful treatment. Notably, it has been found that even though the frequency of HCV-related HCC decreases after achieving a SVR with DAA therapy, the risk of HCC development still remains relatively high [199, 200]. Several studies have suggested that HCV-induced changes to the epigenome persist even after successful treatment with DAAs, potentially contributing to the increased risk of HCC following cure [201–203]. These investigations clearly show that HCV infection has a significant effect on the location of histone modifications across the genome, which significantly alters the host cells’ patterns of gene expression. Surprisingly, these modifications were encoded in the epigenome and continue even after treatment by DAA therapy. There are still unanswered questions about the epigenetic processes that lead to T cell exhaustion and whether it is possible to reprogram the exhausted cell state after curative treatment. Yates and colleagues (2021) demonstrated that the epigenetic scars of T cell exhaustion persist after DAA therapy and continue for an extended period after the infection has been cured**.** Additionally**,** epigenetic scars in TOX and HIF1α persist, along with high expression of neighboring genes after DAA therapy [95]. Strikingly, a recent study investigates the link between chronic HCV infection and biological age acceleration, and if this can be reversed after DAA therapy and HCV elimination. They examined DNA methylation status to estimate an individual’s biological age. The study found that HCV infection accelerated the epigenetic aging process, and this effect was partially reversed after DAA treatment and HCV elimination. However, this reversal was not observed in individuals who developed HCC after HCV elimination [204].

In contrast to genetic alterations, epigenetic modifications are reversible and have a greater impact on gene expression than genetic changes. In a study evaluating the reversal of epigenetic signatures, researchers found that the epigenetic signature was reversed in vitro by drugs that inhibit epigenetic modifying enzymes and by the epidermal growth factor receptor (EGFR) inhibitor, Erlotinib [203]. Consequentially, further research is needed to pinpoint specific upstream, druggable targets where molecular pathways are activated by HCV. This can potentially alter the epigenome and transcriptome. Thus, it will help in developing personalized interventions for the prevention of HCC specifically related to HCV etiology.

## Simulation predictive modelling and machine learning in DAA therapy in HCV management

Achieving complete elimination of HCV at the population level requires addressing individual treatment failures and understanding the complex interactions between virological, host immune, and treatment factors. With the increasing use of DAAs and emerging evidence of resistance, a comprehensive understanding of viral kinetics, immune responses, drug dynamics, and patient variability is essential for developing accurate predictive models and effective intervention strategies. The integration of simulation modeling and machine learning (ML) in HCV management shows promise in optimizing therapeutic approaches and improving treatment outcomes, marking a significant step forward in personalized medicine. Several studies have highlighted the effectiveness of ML models in predicting SVR or treatment failure following DAA therapy. ML techniques, such as support vector machines (SVMs), have demonstrated a 95% accuracy rate in analyzing full-length HCV genome sequences to identify RASs [205]. Moreover, the application of ML algorithms like random forest, XGBoost, and artificial neural networks (ANNs) to large datasets effectively analyzes predictors such as adherence and cirrhosis, underscoring the scalability of ML approaches in clinical research [206]. In the realm of simulation modeling, the focus has traditionally been on analyzing viral dynamics, the emergence of resistance, and the outcomes of treatments through approaches grounded in pharmacokinetics and pharmacodynamics. Adiwijaya et al. introduced a mathematical framework that incorporates the influence of pharmacokinetics and resistance variants, forecasting the probability of achieving SVR based on initial resistance levels and dynamic changes during DAA therapy [207]. Furthering this approach, Ke et al. enriched the model by integrating data on temporal adherence patterns, enhancing the ability to predict resistance emergence and tailor dosing strategies effectively [208].

Furthermore, effective prediction modelling through multivariate logistic regression can be effectively utilized to identify the minimal set of independent variables necessary for predicting the treatment outcomes. An important factor in determining infection and treatment outcome is the strength of the early innate immune response, particularly the induction of type III interferons. By incorporating additional factors such as age, sex, body mass index (BMI), viral load, fibrosis stage, ISG expression, and amino acid substitutions in viral proteins, prediction models can accomplish an ROC AUC approaching 0.85 [209–211]. A recent study has revealed that that being male, older age, elevated ALT and AFP, a high HCV viral load, and the presence of the T risk allele of IL28B rs12979860 were associated with resistance to DAAs, with an accuracy of 85.5% [92].

Despite these advances, immune system dynamics need to be more prominently featured in these models as they influence treatment outcomes and the risk of relapse, especially in populations with compromised immunity. Future research should prioritize the development of hybrid machine learning-simulation frameworks that incorporate immune responses, resistance patterns, and treatment adherence to tailor personalized DAA regimens and advance global elimination efforts.

## Conclusion and future perspective

Despite breakthroughs in DAA therapy for controlling HCV, it does not protect against re-infection. Existing evidence has shown a limited recovery of T cell immunity following DAA treatment, suggesting that a full recovery, similar to that observed following spontaneous viral clearance, might not be attained. This supports that prolonged exposure to antigens leads to permanent alterations in the T cell population. Therefore, it is essential to comprehend the mechanisms of immune dysfunction induced by HCV and the obstacles to immune recovery after clearing the virus to prevent the lasting consequences of chronic HCV infection. Proper modulation of both the humoral and cell-mediated immune response could be an effective strategy for viral eradication.

With the rapid development of modern technologies such as clustered regularly interspaced short palindromic repeat/ CRISPR-associated sequences (CRISPR/Cas) mediated genome editing, a new approach for patients with HCV who are likely to reinfection or do not respond to current treatments is possible. It promises a new tool to downregulate the single-stranded RNA (ssRNA). A group of researchers used the CRISPR-Cas13a system to target the highly conserved internal-ribosomal entry site (IRES) of HCV RNA as a novel antiviral target [212].

Furthermore, it has been suggested that constructing chimeric antigen receptor (CAR) T cells targeting HCV E2 glycoprotein (HCV/E2), the main target of the host humoral immune response and the major variable viral proteins, could be an alternative strategy for patients unresponsive to available therapies [213]. Sautto and colleagues (2016) observed that anti-HCV/E2 CAR T cells can lyse HCV/E2-expressing cells and HCV-infected hepatocytes [214]. This study demonstrates the potential of using CAR T cells as a treatment option for patients with HCV infection who do not respond to current treatments. This approach could provide a new avenue for managing HCV infections and may offer hope for patients who have limited treatment options. Future research and clinical trials are needed to validate the effectiveness and safety of this approach, but the initial results are promising.

Furthermore, investigating the possible synergistic effects of immunomodulatory therapies and DAAs may open up a new window for combined therapeutic approaches. As research advances, a deeper understanding of the intricate immunological landscape during DAA therapy for HCV will not only enhance treatment strategies but also contribute to our broader comprehension of antiviral immunity. Therefore, targeting dysfunctional signaling and remodeling of epigenetic scars can effectively reverse T cell exhaustion. This may have a positive impact on HCV infection, particularly in patients who do not respond to DAA and still need effective therapies. It may also benefit other chronic diseases, such as cancer, which exhibit T cell dysfunction due to prolonged CD8 + T cell stimulation.

## Data Availability

Data sharing is not applicable to this article as no datasets were generated or analyzed during the current study.

## References

[1] Yang J, Qi JL, Wang XX, Li XH, Jin R, Liu BY, et al. The burden of hepatitis C virus in the world, China, India, and the United States from 1990 to 2019. Front public Heal. 2023;11:1041201. 10.3389/fpubh.2023.1041201 PMC10018168 36935711

[2] (WHO) WHO. WHO. Hepatitis C [Internet]. 2024. Available from: https://www.who.int/news-room/fact-sheets/detail/hepatitis-c

[3] Ezzat R, Eltabbakh M, El Kassas M. Unique situation of hepatocellular carcinoma in Egypt: a review of epidemiology and control measures. World J Gastrointest Oncol. 2021;13(12):1919–38. 35070033 10.4251/wjgo.v13.i12.1919 PMC8713321

[4] Stanaway JD, Flaxman AD, Naghavi M, Fitzmaurice C, Vos T, Abubakar I, et al. The global burden of viral hepatitis from 1990 to 2013: findings from the Global Burden of Disease Study 2013. Lancet. 2016;388(10049):1081–8. 27394647 10.1016/S0140-6736(16)30579-7 PMC5100695

[5] Stuart JD, Salinas E, Grakoui A. Immune system control of hepatitis C virus infection. Curr Opin Virol. 2021;46:36–44. 33137689 10.1016/j.coviro.2020.10.002 PMC7979439

[6] Klenerman P, Thimme R. T cell responses in hepatitis C: the good, the bad and the unconventional. Gut. 2012;61(8):1226–34. 21873736 10.1136/gutjnl-2011-300620

[7] Götte M, Feld JJ. Direct-acting antiviral agents for hepatitis C: structural and mechanistic insights. Nat Rev Gastroenterol Hepatol. 2016;13(6):338–51. 10.1038/nrgastro.2016.60. 27147491 10.1038/nrgastro.2016.60

[8] Spearman CW, Dusheiko GM, Hellard M, Sonderup M. Hepatitis C. Lancet. 2019;394(10207):1451–66. 31631857 10.1016/S0140-6736(19)32320-7

[9] Manns MP, Buti M, Gane E, Pawlotsky JM, Razavi H, Terrault N, et al. Hepatitis C virus infection. Nat Rev Dis Prim. 2017;3(1):1–19. 10.1038/nrdp.2017.6 28252637

[10] World Health Organization. Global hepatitis report 2017 [Internet]. 2017 [cited 2023 May 26]. Available from: https://apps.who.int/iris/bitstream/handle/10665/255016/9789?sequence=1

[11] Cooke GS, Andrieux-Meyer I, Applegate TL, Atun R, Burry JR, Cheinquer H, et al. Accelerating the elimination of viral hepatitis: a lancet gastroenterology & hepatology commission. Lancet Gastroenterol Hepatol. 2019;4(2):135–84. 30647010 10.1016/S2468-1253(18)30270-X

[12] Jin F, Dore GJ, Matthews G, Luhmann N, Macdonald V, Bajis S, et al. Prevalence and incidence of hepatitis C virus infection in men who have sex with men: a systematic review and meta-analysis. Lancet Gastroenterol Hepatol. 2021;6(1):39–56. 10.1016/S2468-1253(20)30303-4. 33217341 10.1016/S2468-1253(20)30303-4

[13] Lunemann S, Schlaphoff V, Cornberg M, Wedemeyer H. NK cells in hepatitis C: role in disease susceptibility and therapy. Dig Dis. 2012;30(SUPPL. 1):48–54. 23075868 10.1159/000341680

[14] Tanaka Y, Nishida N, Sugiyama M, Kurosaki M, Matsuura K, Sakamoto N, et al. Genome-wide association of IL28B with response to pegylated interferon-α and ribavirin therapy for chronic hepatitis C. Nat Genet. 2009;41(10):1105–9. 10.1038/ng.449. 19749757 10.1038/ng.449

[15] Ge D, Fellay J, Thompson AJ, Simon JS, Shianna KV, Urban TJ, et al. Genetic variation in IL28B predicts hepatitis C treatment-induced viral clearance. Nature. 2009;461(7262):399–401. 19684573 10.1038/nature08309

[16] Suppiah V, Moldovan M, Ahlenstiel G, Berg T, Weltman M, Abate ML, et al. *IL28B* is associated with response to chronic hepatitis C interferon-α and ribavirin therapy. Nat Genet. 2009;41(10):1100–4. 19749758 10.1038/ng.447

[17] Li K, Foy E, Ferreon JCM, Nakamura M, Ferreon ACM, Ikeda M, et al. Immune evasion by hepatitis C virus NS3/4A protease-mediated cleavage of the Toll-like receptor 3 adaptor protein TRIF. Proc Natl Acad Sci U S A. 2005;102(8):2992–7. 15710891 10.1073/pnas.0408824102 PMC548795

[18] Rehermann B. Hepatitis C virus versus innate and adaptive immune responses: a tale of coevolution and coexistence. J Clin Invest. 2009;119:1745–54. 19587449 10.1172/JCI39133 PMC2701885

[19] Heim MH, Thimme R. Innate and adaptive immune responses in HCV infections. J Hepatol. 2014;61(1):S14-25. 25443342 10.1016/j.jhep.2014.06.035

[20] Harwood NMK, Golden-Mason L, Cheng L, Rosen HR, Mengshol JA. HCV-infected cells and differentiation increase monocyte immunoregulatory galectin-9 production. J Leukoc Biol. 2016;99(3):495–503. 26475932 10.1189/jlb.5A1214-582R PMC6608045

[21] Ahmed F, Ibrahim A, Cooper CL, Kumar A, Crawley AM. Chronic hepatitis C virus infection impairs M1 macrophage differentiation and contributes to CD8+ T-cell dysfunction. Cells. 2019. 10.3390/cells8040374. 31027182 10.3390/cells8040374 PMC6523920

[22] Bility MT, Nio K, Li F, McGivern DR, Lemon SM, Feeney ER, et al. Chronic hepatitis C infection–induced liver fibrogenesis is associated with M2 macrophage activation. Sci Rep. 2016;6(1):39520. 10.1038/srep39520. 28000758 10.1038/srep39520 PMC5175173

[23] Saha B, Kodys K, Szabo G. Hepatitis C virus-induced monocyte differentiation into polarized m2 macrophages promotes stellate cell activation via TGF-β. Cell Mol Gastroenterol Hepatol. 2016;2(3):302-316.e8. 28090562 10.1016/j.jcmgh.2015.12.005 PMC5042356

[24] Rosen HR, Golden-Mason L. Control of HCV infection by natural killer cells and macrophages. Cold Spring Harb Perspect Med. 2020. 10.1101/cshperspect.a037101. 31871225 10.1101/cshperspect.a037101 PMC7447067

[25] Yu MYW, Bartosch B, Zhang P, Guo ZP, Renzi PM, Shen LM, et al. Neutralizing antibodies to hepatitis C virus (HCV) in immune globulins derived from anti-HCV-positive plasma. Proc Natl Acad Sci U S A. 2004;101(20):7705–10. 15136748 10.1073/pnas.0402458101 PMC419670

[26] Pestka JM, Zeisel MB, Bläser E, Schürmann P, Bartosch B, Cosset FL, et al. Rapid induction of virus-neutralizing antibodies and viral clearance in a single-source outbreak of hepatitis C. Proc Natl Acad Sci U S A. 2007;104(14):6025–30. 17392433 10.1073/pnas.0607026104 PMC1851610

[27] Semmo N, Lucas M, Krashias G, Lauer G. Maintenance of HCV-specific T-cell responses in antibody-deficient patients a decade after early therapy. Blood. 2015;107(11):4570–2. 10.1182/blood-2005-11-4522 16717132

[28] Golubovskaya V, Wu L. Different subsets of T cells, memory, effector functions, and CAR-T immunotherapy. Cancers (Basel). 2016;8(3):36. 26999211 10.3390/cancers8030036 PMC4810120

[29] Sun L, Su Y, Jiao A, Wang X, Zhang B. T cells in health and disease. Signal Transduct Target Ther. 2023. 10.1038/s41392-023-01471-y. 37332039 10.1038/s41392-023-01471-y PMC10277291

[30] Thimme R, Oldach D, Chang KM, Steiger C, Ray SC, Chisari FV. Determinants of viral clearance and persistence during acute hepatitis C virus infection. J Exp Med. 2001;194(10):1395–406. 11714747 10.1084/jem.194.10.1395 PMC2193681

[31] Sung PS, Racanelli V, Cheol SE. CD8 + T-cell responses in acute hepatitis C virus infection. Front Immunol. 2014;5:1–7. 24936203 10.3389/fimmu.2014.00266 PMC4047488

[32] Aberle JH, Formann E, Steindl-munda P, Weseslindtner L, Gurguta C, Perstinger G, et al. Prospective study of viral clearance and CD4 + T-cell response in acute hepatitis C primary infection and reinfection. J Clin Virol. 2006;36(1):24–31. 16483838 10.1016/j.jcv.2005.12.010

[33] Mehta SH, Cox A, Hoover DR, Wang X, Mao Q, Ray S, et al. Protection against persistence of hepatitis C. Lancet. 2002;359(9316):1478–83. 11988247 10.1016/S0140-6736(02)08435-0

[34] Wedemeyer H, He XS, Nascimbeni M, Davis AR, Greenberg HB, Hoofnagle JH, et al. Impaired effector function of hepatitis C virus-specific CD8+ T cells in chronic hepatitis C virus infection. J Immunol. 2002;169(6):3447–58. 12218168 10.4049/jimmunol.169.6.3447

[35] Cerny A, Chisari FV. Pathogenesis of chronic hepatitis C: immunological features of hepatic injury and viral persistence. Hepatology. 1999;30(3):595–601. 10462362 10.1002/hep.510300312

[36] Thimme R, Bukh J, Spangenberg HC, Wieland S, Pemberton J, Steiger C, et al. Viral and immunological determinants of hepatitis C virus clearance, persistence, and disease. Proc Natl Acad Sci U S A. 2002;99(24):15661–8. 12441397 10.1073/pnas.202608299 PMC137773

[37] Lechner F, Wong DKH, Dunbar PR, Chapman R, Chung RT, Dohrenwend P, et al. Analysis of successful immune responses in persons infected with hepatitis C virus. J Exp Med. 2000;191(9):1499–512. 10790425 10.1084/jem.191.9.1499 PMC2213430

[38] Grakoui A, Shoukry NH, Woollard DJ, Han JH, Hanson HL, Ghrayeb J, et al. HCV persistence and immune evasion in the absence of memory T cell help. Science. 2003;302(5645):659–62. 14576438 10.1126/science.1088774

[39] Shoukry NH, Grakoui A, Houghton M, Chien DY, Ghrayeb J, Reimann KA, et al. Memory CD8+ T cells are required for protection from persistent hepatitis C virus infection. J Exp Med. 2003;197(12):1645–55. 12810686 10.1084/jem.20030239 PMC2193956

[40] Kim AY, Kuntzen T, Timm J, Nolan BE, Baca MA, Reyor LL, et al. Spontaneous control of HCV is associated with expression of HLA-B *57 and preservation of targeted epitopes. Gastroenterology. 2011;140(2):686-696.e1. 20875418 10.1053/j.gastro.2010.09.042 PMC3021586

[41] Kuniholm MH, Kovacs A, Gao X, Xue X, Marti D, Thio CL, et al. Specific human leukocyte antigen class I and II alleles associated with hepatitis C virus viremia. Hepatology. 2010;51(5):1514–22. 20169624 10.1002/hep.23515 PMC2946382

[42] McKiernan SM, Hagan R, Curry M, McDonald GSA, Kelly A, Nolan N, et al. Distinct MHC class I and II alleles are associated with hepatitis C viral clearance, originating from a single source. Hepatology. 2004;40(1):108–14. 15239092 10.1002/hep.20261

[43] Gowans EJ, Jones KL, Bharadwaj M, Jackson DC. Prospects for dendritic cell vaccination in persistent infection with hepatitis C virus. J Clin Virol. 2004;30(4):283–90. 15163415 10.1016/j.jcv.2004.03.006 PMC4526278

[44] Le G-G, Vallet S, Payan C, Pivert A, Goudeau A. Genetic diversity of the hepatitis C virus and antiviral therapy. World J Gastroenterol. 2007;13(17):2416–26. 17552024 10.3748/wjg.v13.i17.2416 PMC4146759

[45] Wherry EJ. T cell exhaustion. Nat Immunol. 2011;12(6):492–9. 21739672 10.1038/ni.2035

[46] Pauken KE, Wherry EJ. Overcoming T cell exhaustion in infection and cancer. Trends Immunol. 2015;36(4):265–76. 25797516 10.1016/j.it.2015.02.008 PMC4393798

[47] Penna A, Pilli M, Zerbini A, Orlandini A, Mezzadri S, Sacchelli L, et al. Dysfunction and functional restoration of HCV-specific CD8 responses in chronic hepatitis C virus infection. Hepatology. 2007;45(3):588–601. 17326153 10.1002/hep.21541

[48] Nakamoto N, Kaplan DE, Coleclough J, Li Y, Valiga ME, Kaminski M, et al. Functional Restoration of HCV-Specific CD8 T Cells by PD-1 Blockade Is Defined by PD-1 Expression and Compartmentalization. Gastroenterology. 2008;134(7):1927–37. 18549878 10.1053/j.gastro.2008.02.033 PMC2665722

[49] Blackburn SD, Shin H, Haining WN, Zou T, Workman CJ, Polley A, et al. Coregulation of CD8+ T cell exhaustion by multiple inhibitory receptors during chronic viral infection. Nat Immunol. 2009;10(1):29–37. 19043418 10.1038/ni.1679 PMC2605166

[50] Wherry EJ, Blattman JN, Murali-Krishna K, van der Most R, Ahmed R. Viral persistence alters CD8 T-cell immunodominance and tissue distribution and results in distinct stages of functional impairment. J Virol. 2003;77(8):4911–27. 12663797 10.1128/JVI.77.8.4911-4927.2003 PMC152117

[51] Dyck L, Mills KHG. Immune checkpoints and their inhibition in cancer and infectious diseases. Eur J Immunol. 2017;47(5):765–79. 28393361 10.1002/eji.201646875

[52] Mueller SN, Ahmed R. High antigen levels are the cause of T cell exhaustion during chronic viral infection. Proc Natl Acad Sci U S A. 2009;106(21):8623–8. 19433785 10.1073/pnas.0809818106 PMC2688997

[53] Wherry EJ, Barber DL, Kaech SM, Blattman JN, Ahmed R. Antigen-independent memory CD8 T cells do not develop during chronic viral infection. Proc Natl Acad Sci U S A. 2004;101(45):16004–9. 15505208 10.1073/pnas.0407192101 PMC524220

[54] Paley MA, Kroy DC, Odorizzi PM, Johnnidis JB, Dolfi DV, Barnett BE, Bikoff EK, Robertson EJ, Lauer GM, Reiner SL, Wherry EJ. Progenitor and terminal subsets of CD8+ T cells cooperate to contain chronic viral infection. Science. 2012. 10.1126/science.1229620. 23197535 10.1126/science.1229620 PMC3653769

[55] Wieland D, Kemming J, Schuch A, Emmerich F, Knolle P, Neumann-Haefelin C, et al. TCF1+ hepatitis C virus-specific CD8+ T cells are maintained after cessation of chronic antigen stimulation. Nat Commun. 2017. 10.1038/ncomms15050. 28466857 10.1038/ncomms15050 PMC5418623

[56] Utzschneider DT, Charmoy M, Chennupati V, Pousse L, Ferreira DP, Calderon-Copete S, et al. T cell factor 1-expressing memory-like CD8+ T cells sustain the immune response to chronic viral infections. Immunity. 2016;45(2):415–27. 27533016 10.1016/j.immuni.2016.07.021

[57] Alfei F, Kanev K, Hofmann M, Wu M, Ghoneim HE, Roelli P, et al. TOX reinforces the phenotype and longevity of exhausted T cells in chronic viral infection. Nature. 2019;571(7764):265–9. 10.1038/s41586-019-1326-9. 31207605 10.1038/s41586-019-1326-9

[58] Hensel N, Gu Z, Sagar WD, Jechow K, Kemming J, et al. Memory-like HCV-specific CD8+ T cells retain a molecular scar after cure of chronic HCV infection. Nat Immunol. 2021;22(2):229–39. 10.1038/s41590-020-00817-w. 33398179 10.1038/s41590-020-00817-w

[59] Casazza JP, Betts MR, Picker LJ, Koup RA. Decay kinetics of human immunodeficiency virus-specific CD8 + T cells in peripheral blood after initiation of highly active antiretroviral therapy. J Virol. 2001;75(14):6508–16. 11413318 10.1128/JVI.75.14.6508-6516.2001 PMC114374

[60] Shin H, Blackburn SD, Blattman JN, Wherry EJ. Viral antigen and extensive division maintain virus-specific CD8 T cells during chronic infection. J Exp Med. 2007;204(4):941–9. 17420267 10.1084/jem.20061937 PMC2118542

[61] Semmo N, Klenerman P. CD4+ T cell reponses in hepatitis C virus infection. World J Gastroenterol. 2007;13(36):4831–8. 17828814 10.3748/wjg.v13.i36.4831 PMC4611761

[62] Matloubian M, Concepcion RJ, Ahmed R. CD4+ T cells are required to sustain CD8+ cytotoxic T-cell responses during chronic viral infection. J Virol. 1994;68(12):8056–63. 7966595 10.1128/jvi.68.12.8056-8063.1994 PMC237269

[63] Chen DY, Wolski D, Aneja J, Matsubara L, Robilotti B, Hauck G, et al. Hepatitis C virus-specific CD4+ T cell phenotype and function in different infection outcomes. J Clin Invest. 2020;130(2):768–73. 31904582 10.1172/JCI126277 PMC6994113

[64] Raziorrouh B, Ulsenheimer A, Schraut W, Heeg M, Kurktschiev P, Zachoval R, et al. Inhibitory molecules that regulate expansion and restoration of HCV-specific CD4+ T cells in patients with chronic infection. Gastroenterology. 2011;141(4):1422–31. 10.1053/j.gastro.2011.07.004. 21763239 10.1053/j.gastro.2011.07.004

[65] Ackermann C, Smits M, Woost R, Eberhard JM, Peine S, Kummer S, et al. HCV-specific CD4+ T cells of patients with acute and chronic HCV infection display high expression of TIGIT and other co-inhibitory molecules. Sci Rep. 2019;9(1):1–12. 31337800 10.1038/s41598-019-47024-8 PMC6650447

[66] Sakaguchi S, Miyara M, Costantino CM, Hafler DA. FOXP3 + regulatory T cells in the human immune system. Nat Rev Immunol. 2010;10(7):490–500. 10.1038/nri2785. 20559327 10.1038/nri2785

[67] Williams LM, Rudensky AY. Maintenance of the Foxp3-dependent developmental program in mature regulatory T cells requires continued expression of Foxp3. Nat Immunol. 2007;8(3):277–84. 17220892 10.1038/ni1437

[68] Wildin RS, Ramsdell F, Peake J, Faravelli F, Casanova JLR, Buist N, et al. Table 1 Syndromes caused by mutations of human Fox-protein genes. Proc Natl Acad Sci USA. 2000;66:683–90.

[69] Khattri R, Cox T, Yasayko SA, Ramsdell F. An essential role for Scurfin in CD4+CD25+T regulatory cells. J Immunol. 2017;198(3):993–8. 28115588

[70] Rushbrook SM, Ward SM, Unitt E, Vowler SL, Lucas M, Klenerman P, et al. Regulatory t cells suppress in vitro proliferation of virus-specific CD8 + t cells during persistent hepatitis c virus infection. J Virol. 2005;79(12):7852–9. 15919939 10.1128/JVI.79.12.7852-7859.2005 PMC1143649

[71] Bolacchi F, Sinistro A, Ciaprini C, Demin F, Capozzi M, Carducci FC, et al. Increased hepatitis C virus (HCV)-specific CD4+CD25+ regulatory T lymphocytes and reduced HCV-specific CD4+ T cell response in HCV-infected patients with normal versus abnormal alanine aminotransferase levels. Clin Exp Immunol. 2006;144(2):188–96. 16634790 10.1111/j.1365-2249.2006.03048.x PMC1809656

[72] Ward SM, Fox BC, Brown PJ, Worthington J, Fox SB, Chapman RW, et al. Quantification and localisation of FOXP3+ T lymphocytes and relation to hepatic inflammation during chronic HCV infection. J Hepatol. 2007;47(3):316–24. 17475362 10.1016/j.jhep.2007.03.023

[73] Turovskaya O, Kim G, Cheroutre H, Kronenberg M, Madan R. Interleukin 10 acts on regulatory t cells to maintain expression of the transcription factor Foxp3 and suppressive function in mice with colitis. Nat Immunol. 2009;10(11):1178–84. 19783988 10.1038/ni.1791 PMC2898179

[74] Hirotoshi E, Nobuhiro N, Yun L, Emma G, et al. Identification and in vitro expansion of functional antigen-specific CD25+ FoxP3+ regulatory T cells in hepatitis C virus infection. J Virol. 2008;82(10):5043–53. 10.1128/jvi.01548-07. 18337568 10.1128/JVI.01548-07 PMC2346728

[75] Langhans B, Braunschweiger I, Arndt S, Schulte W, Satoguina J, Layland L, et al. Core-specific adaptive regulatory T-cells in different outcomes of hepatitis C. Clin Sci. 2010;119(2):97–109. 10.1042/CS20090661 20222873

[76] Ji XJ, Ma CJ, Wang JM, Wu XY, Niki T, Hirashima M, et al. Immunomodulatory role of the hepatocyte during HCV infection: driving CD 4 + CD 25 + Foxp 3 + Regulatory T cell Development through the Tim-3 / Gal-9 Pathway. Eur J Immunol. 2013;43(2):458–67. 23161469 10.1002/eji.201242768 PMC3757554

[77] Hall CHT, Kassel R, Tacke RS, Hahn YS. HCV+ hepatocytes induce human regulatory CD4+ T cells through the production of TGF-β. PLoS One. 2010;5(8):e12154. 10.1371/journal.pone.0012154. 20730048 10.1371/journal.pone.0012154 PMC2921368

[78] Hall CHT, Kassel R, Tacke RS, Hahn YS. HCV+ hepatocytes induce human regulatory CD4+ T cells through the production of TGF-beta. PLoS One. 2010;5(8):e12154. 20730048 10.1371/journal.pone.0012154 PMC2921368

[79] Fernandez-Ponce C, Dominguez-Villar M, Aguado E, Garcia-Cozar F. CD4+ primary T cells expressing HCV-core protein upregulate Foxp3 and IL-10, suppressing CD4 and CD8 T cells. PLoS ONE. 2014;9(1):e85191. 24465502 10.1371/journal.pone.0085191 PMC3896374

[80] Liu M, Chen HY, Luo L, Wang Y, Zhang D, Song N, et al. Neutralization of IL-10 produced by B cells promotes protective immunity during persistent HCV infection in humanized mice. Eur J Immunol. 2020;50(9):1350–61. 10.1002/eji.201948488. 32339264 10.1002/eji.201948488

[81] Meng P, Zhao S, Niu X, Fu N, Su S, Wang R, et al. Involvement of the interleukin-23/interleukin-17 axis in chronic hepatitis C virus infection and its treatment responses. Int J Mol Sci. 2016. 10.3390/ijms17071070. 27428948 10.3390/ijms17071070 PMC4964446

[82] Kared H, Fabre T, Bédard N, Bruneau J, Shoukry NH. Galectin-9 and IL-21 mediate cross-regulation between Th17 and Treg cells during acute hepatitis C. PLoS Pathog. 2013;9(6):e1003422. 10.1371/journal.ppat.1003422. 23818845 10.1371/journal.ppat.1003422 PMC3688567

[83] Ahmed HE. The impact of serum interleukin-17 on chronic hepatitis C and its sequelae. J Liver. 2014;03(04):163.

[84] Hao C, Zhou Y, He Y, Fan C, Sun L, Wei X, et al. Imbalance of regulatory T cells and T helper type 17 cells in patients with chronic hepatitis C. Immunology. 2014;143(4):531–8. 10.1111/imm.12330. 24903732 10.1111/imm.12330 PMC4253501

[85] Ji XJ, Ma CJ, Wang JM, Wu XY, Niki T, Hirashima M, et al. HCV-infected hepatocytes drive CD4+CD25+Foxp3+ regulatory T-cell development through the Tim-3/Gal-9 pathway. Eur J Immunol. 2013. 10.1002/eji.201242768. 23161469 10.1002/eji.201242768 PMC3757554

[86] Moorman JP, Wang JM, Zhang Y, Ji XJ, Ma CJ, Wu XY, et al. Tim-3 pathway controls regulatory and effector T cell balance during hepatitis C virus infection. J Immunol. 2012;189(2):755–66. 10.4049/jimmunol.1200162. 22706088 10.4049/jimmunol.1200162 PMC3392408

[87] Nieves-Rosado HM, Banerjee H, Kane LP. Tim-3 deletion on Treg increases virus-specific T cell response and reduces viral burden in chronic LCMV infection. J Immunol. 2022;208(1_Supplement):110.13-110.13. 10.4049/jimmunol.208.Supp.110.13.34819391

[88] Nan Y, Su S, Niu X, Zhao S, Zhang Y, Wang R, et al. Tim-3 suppression combined with TLR3 activation enhances antiviral immune response in patients with chronic HCV infection. J Int Med Res. 2016;44(4):806–16. 10.1177/0300060516647548. 27329385 10.1177/0300060516647548 PMC5536634

[89] Nasab SDM, Vasmehjani AA, Kaghazian H, Mardani R, Zali F, Ahmadi N, et al. Association of IL28B (IFNL3) rs12979860 mRNA levels, viral load, and liver function among HCV genotype 1a patients. Gastroenterol Hepatol from Bed to Bench. 2018;2019(12):S156–62. PMC7011071 32099617

[90] Ragheb MM, Nemr NA, Kishk RM, Mandour MF, Abdou MM, Matsuura K, et al. Strong prediction of virological response to combination therapy by IL28B gene variants rs12979860 and rs8099917 in chronic hepatitis C genotype 4. Liver Int. 2014;34(6):890–5. 24102823 10.1111/liv.12321

[91] Balagopal A, Thomas DL, Thio CL. IL28B and the control of hepatitis C virus infection. Gastroenterology. 2010;139:1865–76. 10.1053/j.gastro.2010.10.004. 20950615 10.1053/j.gastro.2010.10.004 PMC3072961

[92] Abdelaziz AI, Abdelsameea E, Abdel-Samiee M, Ghanem SE, Wahdan SA, Elsherbiny DA, et al. Effect of immunogenetics polymorphism and expression on direct-acting antiviral drug response in chronic hepatitis C. Clin Exp Med. 2024;24(1):184. 39117877 10.1007/s10238-024-01432-x PMC11310263

[93] Moran-Salvador E, Mann J. Epigenetics and liver fibrosis. Cmgh. 2017;4(1):125–34. 10.1016/j.jcmgh.2017.04.007. 28593184 10.1016/j.jcmgh.2017.04.007 PMC5453904

[94] Feng Y, Arvey A, Chinen T, Van Der Veeken J, Gasteiger G, Rudensky AY. Control of the inheritance of regulatory T cell identity by a cis element in the foxp3 locus. Cell. 2014;158(4):749–63. 25126783 10.1016/j.cell.2014.07.031 PMC4151558

[95] Yates KB, Tonnerre P, Martin GE, Gerdemann U, Al Abosy R, Comstock DE, et al. Epigenetic scars of CD8+ T cell exhaustion persist after cure of chronic infection in humans. Nat Immunol. 2021;22(8):1020–9. 10.1038/s41590-021-00979-1. 34312547 10.1038/s41590-021-00979-1 PMC8600539

[96] Zediak VP, Johnnidis JB, Wherry EJ, Berger SL. persistently open chromatin at effector gene loci in resting memory CD8+ T cells independent of transcriptional status. J Immunol. 2011;186(5):2705–9. 21278341 10.4049/jimmunol.1003741 PMC3474546

[97] Wedemeyer H, Khera T, Strunz B, Björkström NK. Reversal of immunity after clearance of chronic HCV infection-all reset? Front Immunol. 2020;11:571166. 33133084 10.3389/fimmu.2020.571166 PMC7578424

[98] Winkler F, Hipp AV, Ramirez C, Martin B, Villa M, Neuwirt E, et al. Enolase represents a metabolic checkpoint controlling the differential exhaustion programmes of hepatitis virus-specific CD8 + T cells. Gut. 2023;72(10):1971–84. 37541771 10.1136/gutjnl-2022-328734 PMC10511960

[99] Pawlotsky JM, Feld JJ, Zeuzem S, Hoofnagle JH. From non-A, non-B hepatitis to hepatitis C virus cure. J Hepatol. 2015;62(S1):S87-99. 10.1016/j.jhep.2015.02.006. 25920094 10.1016/j.jhep.2015.02.006

[100] Geddawy A, Ibrahim YF, Elbahie NM, Ibrahim MA. Direct acting anti-hepatitis C virus drugs: clinical pharmacology and future direction. J Transl Intern Med. 2017;5(1):8–17. 10.1515/jtim-2017-0007 PMC5490957 28680834

[101] Meewan I, Zhang X, Roy S, Ballatore C, O’Donoghue AJ, Schooley RT, et al. Discovery of new inhibitors of hepatitis C virus NS3/4A protease and its D168A mutant. ACS Omega. 2019;4(16):16999–7008. 31646247 10.1021/acsomega.9b02491 PMC6796237

[102] Zhang X. Direct anti-HCV agents. Acta Pharm Sin B. 2016;6(1):26–31. 10.1016/j.apsb.2015.09.008. 26904396 10.1016/j.apsb.2015.09.008 PMC4724659

[103] Calvaruso V, Mazzarelli C, Milazzo L, Badia L, Pasulo L, Guaraldi G, et al. Daclatasvir-based regimens in HCV cirrhosis: experience from the Italian early access program. Sci Rep. 2019;9(1):1–8. 30679515 10.1038/s41598-018-36734-0 PMC6345835

[104] Bartenschlager R, Baumert TF, Bukh J, Houghton M, Lemon SM, Lindenbach BD, et al. Critical challenges and emerging opportunities in hepatitis C virus research in an era of potent antiviral therapy: considerations for scientists and funding agencies. Virus Res. 2018;248:53–62. 10.1016/j.virusres.2018.02.016. 29477639 10.1016/j.virusres.2018.02.016

[105] Jiang H, Wang X, Luo B, Cong X, Jin Q, Qin H, et al. Direct antiviral agents upregulate natural killer cell potential activity in chronic hepatitis C patients. Clin Exp Med. 2019;19(3):299–308. 10.1007/s10238-019-00564-9. 31218578 10.1007/s10238-019-00564-9

[106] Llorens-Revull M, Costafreda MI, Rico A, Guerrero-Murillo M, Soria ME, Píriz-Ruzo S, et al. Partial restoration of immune response in Hepatitis C patients after viral clearance by direct-acting antiviral therapy. PLoS One. 2021;16(7):1–18. 10.1371/journal.pone.0254243 PMC8270431 34242330

[107] Meissner EG, Wu D, Osinusi A, Bon D, Virtaneva K, Sturdevant D, et al. Endogenous intrahepatic IFNs and association with IFN-free HCV treatment outcome. J Clin Invest. 2014;124(8):3352–63. 24983321 10.1172/JCI75938 PMC4109554

[108] Alao H, Cam M, Keembiyehetty C, Zhang F, Serti E, Suarez D, et al. Baseline intrahepatic and peripheral innate immunity are associated with hepatitis C virus clearance during direct-acting antiviral therapy. Hepatology. 2018;68(6):2078–88. 29704252 10.1002/hep.29921 PMC6204120

[109] Serti E, Chepa-lotrea X, Kim YJ, Keane M, Fryzek N, Liang J, et al. Successful interferon-free therapy of chronic hepatitis C virus infection normalizes natural killer cell function. Gastroenterology. 2015;149(1):190–200. 25754160 10.1053/j.gastro.2015.03.004 PMC4523392

[110] Liang TJ, Ghany M, Rehermann B, Serti E, Park H, Keane M, et al. Rapid decrease in hepatitis C viremia by direct acting antivirals improves the natural killer cell response to IFNα. Gut. 2017;66(4):724–35. 26733671 10.1136/gutjnl-2015-310033 PMC6886885

[111] Strunz B, Hengst J, Deterding K, Manns MP, Cornberg M, Ljunggren HG, et al. Chronic hepatitis C virus infection irreversibly impacts human natural killer cell repertoire diversity. Nat Commun. 2018. 10.1038/s41467-018-04685-9. 29891939 10.1038/s41467-018-04685-9 PMC5995831

[112] Howson LJ, Salio M, Cerundolo V. MR1-restricted mucosal-associated invariant T cells and their activation during infectious diseases. Front Immunol. 2015;6:303. 26136743 10.3389/fimmu.2015.00303 PMC4468870

[113] Bolte FJ, O’Keefe AC, Webb LM, Serti E, Rivera E, Liang TJ, et al. Intra-hepatic depletion of mucosal-associated invariant T cells in hepatitis C virus-induced liver inflammation. Gastroenterology. 2017;153(5):1392-1403.e2. 28780074 10.1053/j.gastro.2017.07.043 PMC5669813

[114] Hengst J, Strunz B, Deterding K. Nonreversible MAIT cell-dysfunction in chronic hepatitis C virus infection despite successful interferon-free therapy. Transplantation. 2016;100(11):2237–8. 10.1002/eji.201646447 27296288

[115] Spaan M, Hullegie SJ, Beudeker BJB, Kreefft K, Van Oord GW, Groothuismink ZMA, et al. Frequencies of circulating MAIT cells are diminished in chronic hCV, HIV and HCV/ HIV Co-Infection and do not recover during therapy. PLoS ONE. 2016;11(7):1–13. 10.1371/journal.pone.0159243 PMC4945024 27416100

[116] Hengst J, Falk CS, Schlaphoff V, Deterding K, Manns MP, Cornberg M, et al. Direct-acting antiviral-induced hepatitis C virus clearance does not completely restore the altered cytokine and chemokine milieu in patients with chronic hepatitis C. J Infect Dis. 2016;214(12):1965–74. 27683821 10.1093/infdis/jiw457

[117] Barnes E, Gelderblom HC, Humphreys I, Semmo N, Reesink HW, Beld MGHM, et al. Cellular immune responses during high-dose interferon-α induction therapy for hepatitis C virus infection. J Infect Dis. 2009;199(6):819–28. 10.1086/597072. 19434929 10.1086/597072

[118] Missale G, Pilli M, Zerbini A, Penna A, Ravanetti L, Barili V, et al. Lack of full CD8 functional restoration after antiviral treatment for acute and chronic hepatitis C virus infection. Gut. 2012;61(7):1076–84. 22337949 10.1136/gutjnl-2011-300515

[119] Larrubia JR, Moreno-Cubero E, Miquel J, Sanz-de-Villalobos E. Hepatitis C virus-specific cytotoxic T cell response restoration after treatment-induced hepatitis C virus control. World J Gastroenterol. 2015;21(12):3480–91. 25834312 10.3748/wjg.v21.i12.3480 PMC4375569

[120] Luxenburger H, Neumann-Haefelin C, Thimme R, Boettler T. HCV-specific T cell responses during and after chronic HCV infection. Viruses. 2018. 10.3390/v10110645. 30453612 10.3390/v10110645 PMC6265781

[121] Sidharthan S, Kohli A, Sims Z, Nelson A, Osinusi A, Masur H, et al. Utility of hepatitis C viral load monitoring on direct-acting antiviral therapy. Clin Infect Dis an Off Publ Infect Dis Soc Am. 2015;60(12):1743–51. 10.1093/cid/civ170 PMC4834854 25733369

[122] Maasoumy B, Buggisch P, Mauss S, Boeker KHW, Müller T, Günther R, et al. Clinical significance of detectable and quantifiable HCV RNA at the end of treatment with ledipasvir/sofosbuvir in GT1 patients. Liver Int Off J Int Assoc Study Liver. 2018;38(11):1906–10. 10.1111/liv.13932 30022590

[123] Shrivastava S, Bhatta M, Ward H, Romani S, Lee R, Rosenthal E, et al. Multitarget direct-acting antiviral therapy is associated with superior immunologic recovery in patients coinfected with human immunodeficiency virus and hepatitis C virus. Hepatol Commun. 2018;2(12):1451–66. 30556035 10.1002/hep4.1258 PMC6287478

[124] Burchill MA, Golden-Mason L, Wind-Rotolo M, Rosen HR. Memory re-differentiation and reduced lymphocyte activation in chronic HCV-infected patients receiving direct-acting antivirals. J Viral Hepat. 2015;22(12):983–91. 26482547 10.1111/jvh.12465

[125] Meissner EG, Kohli A, Higgins J, Lee YJ, Prokunina O, Wu D, et al. Rapid changes in peripheral lymphocyte concentrations during interferon-free treatment of chronic hepatitis C virus infection. Hepatol Commun. 2017;1(7):586–94. 29202115 10.1002/hep4.1074 PMC5703427

[126] Najafi Fard S, Schietroma I, Corano Scheri G, Giustini N, Serafino S, Cavallari EN, et al. Direct-acting antiviral therapy enhances total CD4+ and CD8+ T-cells responses, but does not alter T-cells activation among HCV mono-infected, and HCV/HIV-1 co-infected patients. Clin Res Hepatol Gastroenterol. 2018;42(4):319–29. 10.1016/j.clinre.2017.11.006. 29279268 10.1016/j.clinre.2017.11.006

[127] Langhans B, Nischalke HD, Krämer B, Hausen A, Dold L, van Heteren P, et al. Increased peripheral CD4+ regulatory T cells persist after successful direct-acting antiviral treatment of chronic hepatitis C. J Hepatol. 2017;66(5):888–96. 10.1016/j.jhep.2016.12.019. 28040549 10.1016/j.jhep.2016.12.019

[128] Vranjkovic A, Deonarine F, Kaka S, Angel JB, Cooper CL, Crawley AM. Direct-acting antiviral treatment of HCV infection does not resolve the dysfunction of circulating CD8+ T-cells in advanced liver disease. Front Immunol. 2019;10(AUG):1–18. 31456810 10.3389/fimmu.2019.01926 PMC6700371

[129] Reig M, Boix L, Bruix J. The impact of direct antiviral agents on the development and recurrence of hepatocellular carcinoma. Liver Int. 2016;2017(37):136–9. 10.1111/liv.13321 28052619

[130] Nyberg AH. The association of extrahepatic cancers with chronic hepatitis. Adv Hepatol. 2016;12(3):185–7. PMC4872847 27231448

[131] Houghton M. Prospects for prophylactic and therapeutic vaccines against the hepatitis C viruses. Immunol Rev. 2011;239(1):99–108. 21198667 10.1111/j.1600-065X.2010.00977.x

[132] Martin B, Hennecke N, Lohmann V, Kayser A, Neumann-Haefelin C, Kukolj G, et al. Restoration of HCV-specific CD8+ T cell function by interferon-free therapy. J Hepatol. 2014;61(3):538–43. 10.1016/j.jhep.2014.05.043. 24905492 10.1016/j.jhep.2014.05.043

[133] Perpiñán E, Pérez-Del-Pulgar S, Londoño MC, Mariño Z, Lens S, Leonel T, et al. Chronic genotype 1 hepatitis C along with cirrhosis drives a persistent imprint in virus-specific CD8+ T cells after direct-acting antiviral therapies. J Viral Hepat. 2020;27(12):1408–18. 32812325 10.1111/jvh.13370

[134] Tonnerre P, Wolski D, Subudhi S, Aljabban J, Hoogeveen RC, Damasio M, et al. Differentiation of exhausted CD8+ T cells after termination of chronic antigen stimulation stops short of achieving functional T cell memory. Nat Immunol. 2021;22(8):1030–41. 10.1038/s41590-021-00982-6. 34312544 10.1038/s41590-021-00982-6 PMC8323980

[135] Callendret B, Eccleston HB, Satterfield W, Capone S, Folgori A, Cortese R, et al. Persistent hepatitis C viral replication despite priming of functional CD8+ T cells by combined therapy with a vaccine and a direct-acting antiviral. Hepatology. 2016;63(5):1442–54. 26513111 10.1002/hep.28309 PMC4840073

[136] Han JW, Sung PS, Kim KH, Hong SH, Shin EC, Jun Song M, et al. Dynamic changes in ex vivo T-cell function after viral clearance in chronic HCV infection. J Infect Dis. 2019;220(8):1290–301. 31152667 10.1093/infdis/jiz291

[137] Raziorrouh B, Sacher K, Tawar RG, Emmerich F, Neumann-Haefelin C, Baumert TF, et al. Virus-specific CD4+ T cells have functional and phenotypic characteristics of follicular T-helper cells in patients with acute and chronic HCV infections. Gastroenterology. 2016;150(3):696–706. 10.1053/j.gastro.2015.11.005. 26584604 10.1053/j.gastro.2015.11.005

[138] Smits M, Zoldan K, Ishaque N, Gu Z, Jechow K, Wieland D, et al. Follicular T helper cells shape the HCV-specific CD4+ T cell repertoire after virus elimination. J Clin Invest. 2020;130(2):998–1009. 31697649 10.1172/JCI129642 PMC6994124

[139] Burchill MA, Salomon MP, Golden-Mason L, Wieland A, Maretti-Mira AC, Gale M, et al. Single-cell transcriptomic analyses of T cells in chronic HCV-infected patients dominated by DAA-induced interferon signaling changes. PLoS Pathog. 2021;17(8):1–18. 10.1371/journal.ppat.1009799. 10.1371/journal.ppat.1009799 PMC8376199 34370798

[140] Serrano-Villar S, Sainz T, Lee SA, Hunt PW, Sinclair E, Shacklett BL, et al. HIV-infected individuals with low CD4/CD8 ratio despite effective antiretroviral therapy exhibit altered T cell subsets, heightened CD8+ T cell activation, and increased risk of non-AIDS morbidity and mortality. PLoS Pathog. 2014;10(5):e1004078. 24831517 10.1371/journal.ppat.1004078 PMC4022662

[141] El Menshawy N, Hassan N, Khariza M, AlAshery H, Baghat M, Ashour R. CD4/CD8 ratio could be predictor of burden hepatocellular carcinoma in Egyptian chronic hepatitis C after combined sofosbuvir and daclatasvir therapy. Afr Health Sci. 2023;23(1):198–212. 37545943 10.4314/ahs.v23i1.22 PMC10398471

[142] Rossotti R, Merli M, Baiguera C, Bana NB, Rezzonico LF, D’Amico F, et al. Impact of treatment with direct-acting antivirals on inflammatory markers and autoantibodies in HIV/HCV co-infected individuals. J Viral Hepat. 2023;30(6):530–9. 36773329 10.1111/jvh.13818

[143] Fang Q, Deng Y, Liang R, Mei Y, Hu Z, Wang J, et al. CD19+CD24hiCD38hi regulatory B cells: a potential immune predictive marker of severity and therapeutic responsiveness of hepatitis C. Am J Transl Res. 2020;12(3):889–900. 32269721 PMC7137049

[144] Wu SF, Tseng CW, Ho YC, Chen YC, Ko PH, He YT, et al. Regulatory t cell function modulated after successful direct-acting antiviral treatment for chronic hepatitis c patients. Dig Dis Sci. 2020;65(5):1385–95. 10.1007/s10620-019-05850-w. 31559553 10.1007/s10620-019-05850-w

[145] Cai H, Liu G, Zhong J, Zheng K, Xiao H, Li C, et al. Immune checkpoints in viral infections. Viruses. 2020;12(9):1–23. 10.3390/v12091051 PMC7551039 32967229

[146] Mackie K. Coregulation of CD8+ T cell exhaustion during chronic viral infection by multiple inhibitory receptors. Physiol Behav. 2017;176(1):139–48.28363838

[147] Kole A, Maloy KJ. Control of intestinal inflammation by interleukin-10. Vol. 380, Current Topics in Microbiology and Immunology. 2014. 19–38 p. 10.1007/978-3-662-43492-5_2 25004812

[148] Nakamoto N, Kaplan DE, Coleclough J, Li Y, Valiga ME, Kaminski M, et al. Functional restoration of HCV-specific CD8 T-cells by PD1 blockade is defined by PD1 expression and compartmentalization. Gastroenterology. 2008;134(7):1927–37. 18549878 10.1053/j.gastro.2008.02.033 PMC2665722

[149] Barber DL, Wherry EJ, Masopust D, Zhu B, Allison JP, Sharpe AH, et al. Restoring function in exhausted CD8 T cells during chronic viral infection. Nature. 2006;439(7077):682–7. 16382236 10.1038/nature04444

[150] Freeman GJ, Wherry EJ, Ahmed R, Sharpe AH. Reinvigorating exhausted HIV-specific T cells via PD-1-PD-1 ligand blockade. J Exp Med. 2006;203(10):2223–7. 17000870 10.1084/jem.20061800 PMC2118103

[151] Perelló MC, Fernández-Carrillo C, Londoño MC, Arias-Loste T, Hernández-Conde M, Llerena S, et al. Reactivation of herpesvirus in patients with hepatitis C treated with direct-acting antiviral agents. Clin Gastroenterol Hepatol Off Clin Pract J Am Gastroenterol Assoc. 2016;14(11):1662-1666.e1. 10.1016/j.cgh.2016.05.016 27211502

[152] El Kassas M, Funk AL, Salaheldin M, Shimakawa Y, Eltabbakh M, Jean K, et al. Increased recurrence rates of hepatocellular carcinoma after DAA therapy in a hepatitis C-infected Egyptian cohort: a comparative analysis. J Viral Hepat. 2018;25(6):623–30. 29274197 10.1111/jvh.12854

[153] Waziry R, Hajarizadeh B, Grebely J, Amin J, Law M, Danta M, et al. Hepatocellular carcinoma risk following direct-acting antiviral HCV therapy: a systematic review, meta-analyses, and meta-regression. J Hepatol. 2017;67(6):1204–12. 28802876 10.1016/j.jhep.2017.07.025

[154] Li S, Mizukoshi E, Kawaguchi K, Miura M, Nishino M, Shimakami T, et al. Alterations in hepatocellular carcinoma-specific immune responses following Hepatitis C virus elimination by direct-acting antivirals. Int J Mol Sci. 2022. 10.3390/ijms231911623. 36232928 10.3390/ijms231911623 PMC9570039

[155] Kober J, Leitner J, Klauser C, Woitek R, Majdic O, Stöckl J, et al. The capacity of the TNF family members 4–1BBL, OX40L, CD70, GITRL, CD30L and LIGHT to costimulate human T cells. Eur J Immunol. 2008;38(10):2678–88. 18825741 10.1002/eji.200838250 PMC2975061

[156] Moreno-Cubero E, Subirá D, Sanz-de-Villalobos E, Parra-Cid T, Madejón A, Miquel J, et al. According to hepatitis C virus (HCV) infection stage, interleukin-7 Plus 4–1BB triggering alone or combined with PD-1 blockade increases TRAF1(low) HCV-specific CD8(+) cell reactivity. J Virol. 2018;92(2):1–23. 10.1128/JVI.01443-17 PMC5752940 29093082

[157] Wang C, McPherson AJ, Jones RB, Kawamura KS, Lin GHY, Lang PA, et al. Loss of the signaling adaptor TRAF1 causes CD8+ T cell dysregulation during human and murine chronic infection. J Exp Med. 2012;209(1):77–91. 22184633 10.1084/jem.20110675 PMC3260874

[158] Wang C, McPherson AJ, Jones RB, Kawamura KS, Lin GHY, Lang PA, et al. Loss of the signaling adaptor TRAF1 causes CD8+ T cell dysregulation during human and murine chronic infection. J Exp Med. 2011;209(1):77–91. 10.1084/jem.20110675. 22184633 10.1084/jem.20110675 PMC3260874

[159] Salem ML, El-Badawy A. Programmed death-1/programmed death-L1 signaling pathway and its blockade in hepatitis C virus immunotherapy. World J Hepatol. 2015;7(23):2449–58. 26483866 10.4254/wjh.v7.i23.2449 PMC4606200

[160] Fuller MJ, Callendret B, Zhu B, Freeman GJ, Hasselschwert DL, Satterfield W, et al. Immunotherapy of chronic hepatitis C virus infection with antibodies against programmed cell death-1 (PD-1). Proc Natl Acad Sci U S A. 2013;110(37):15001–6. 23980172 10.1073/pnas.1312772110 PMC3773803

[161] Gardiner D, Lalezari J, Lawitz E, DiMicco M, Ghalib R, Reddy KR, et al. A randomized, double-blind, placebo-controlled assessment of BMS-936558, a fully human monoclonal antibody to programmed death-1 (PD-1), in patients with chronic hepatitis C virus infection. PLoS One. 2013;8(5):e63818. 23717490 10.1371/journal.pone.0063818 PMC3661719

[162] Romani S, Stafford K, Nelson A, Bagchi S, Kottilil S, Poonia B. Peripheral PD-1+ T cells co-expressing inhibitory receptors predict SVR with ultra short duration DAA therapy in HCV infection. Front Immunol. 2019;10(JUN):1–12. 31316516 10.3389/fimmu.2019.01470 PMC6610534

[163] Osegueda A, Polo ML, Baquero L, Urioste A, Ghiglione Y, Paz S, et al. Markers of natural killer cell exhaustion in HIV/HCV coinfection and their dynamics after HCV clearance mediated by direct-acting antivirals. Open Forum Infect Dis. 2023;10(12):1–11. 10.1093/ofid/ofad591. 10.1093/ofid/ofad591 PMC10723816 38107019

[164] Shive CL, Kowal CM, Desotelle AF, Nguyen Y, Carbone S, Kostadinova L, et al. Endotoxemia associated with liver disease correlates with systemic inflammation and T cell exhaustion in hepatitis C virus infection. Cells. 2023. 10.3390/cells12162034. 37626844 10.3390/cells12162034 PMC10453378

[165] Bi W, Kraft A, Engelskircher S, Mischke J, Witte M, Klawonn F, et al. Proteomics reveals a global phenotypic shift of NK cells in HCV patients treated with direct-acting antivirals. Eur J Immunol. 2023;53(11):1–22. 10.1002/eji.202250291 37515498

[166] Ramadan HKA, Badr G, Ramadan NK, Sayed A. Enhanced immune responses, pi3k/akt and jak/stat signaling pathways following hepatitis c virus eradication by direct-Acting antiviral therapy among Egyptian patients: a case control study. Pathog Dis. 2021;79(3):1–11. 10.1093/femspd/ftab008 33524139

[167] Szereday L, Meggyes M, Berki T, Miseta A, Farkas N, Gervain J, et al. Direct-acting antiviral treatment downregulates immune checkpoint inhibitor expression in patients with chronic hepatitis C. Clin Exp Med. 2020;20(2):219–30. 10.1007/s10238-020-00618-3. 32108916 10.1007/s10238-020-00618-3 PMC7181552

[168] Raimondo G, Brunetto MR, Pontisso P, Smedile A, Maina AM, Saitta C, et al. Longitudinal evaluation reveals a complex spectrum of virological profiles in hepatitis B virus/hepatitis C virus-coinfected patients. Hepatology. 2006;43(1):100–7. 16323213 10.1002/hep.20944

[169] Wiegand SB, Jaroszewicz J, Potthoff A, Höner zu Siederdissen C, Maasoumy B, Deterding K, et al. Dominance of hepatitis C virus (HCV) is associated with lower quantitative hepatitis B surface antigen and higher serum interferon-γ-induced protein 10 levels in HBV/HCV-coinfected patients. Clin Microbiol Infect. 2015;21(7):710.e1-710.e9. 10.1016/j.cmi.2015.03.003. 25882360 10.1016/j.cmi.2015.03.003

[170] Bersoff-Matcha SJ, Cao K, Jason M, Ajao A, Jones SC, Meyer T, et al. Hepatitis B virus reactivation associated with direct-acting antiviral therapy for chronic hepatitis C virus: a review of cases reported to the U.S. food and drug administration adverse event reporting system. Ann Intern Med. 2017;166(11):792–8. 28437794 10.7326/M17-0377

[171] Chen G, Wang C, Chen J, Ji D, Wang Y, Wu V, et al. Hepatitis B reactivation in hepatitis B and C coinfected patients treated with antiviral agents: a systematic review and meta-analysis. Hepatology. 2017;66(1):13–26. 28195337 10.1002/hep.29109

[172] Yeh ML, Huang CF, Huang CI, Holmes JA, Hsieh MH, Tsai YS, et al. Hepatitis B-related outcomes following direct-acting antiviral therapy in Taiwanese patients with chronic HBV/HCV co-infection. J Hepatol. 2020;73(1):62–71. 10.1016/j.jhep.2020.01.027. 32061869 10.1016/j.jhep.2020.01.027

[173] Azeem HA, Alkabeer AM, Mohammed AS, Hussein AA. Study of hepatitis B virus infection, reactivation among patients with chronic hepatitis C infection treated by direct antiviral agents (DAAs). Egypt Liver J. 2021;11(1):53.

[174] Eyre NS, Phillips RJ, Bowden S, Yip E, Dewar B, Locarnini SA, et al. Hepatitis B virus and hepatitis C virus interaction in Huh-7 cells. J Hepatol. 2009;51(3):446–57. 19596477 10.1016/j.jhep.2009.04.025

[175] Bellecave P, Gouttenoire J, Gajer M, Brass V, Koutsoudakis G, Blum HE, et al. Hepatitis B and C virus coinfection: a novel model system reveals the absence of direct viral interference. Hepatology. 2009;50(1):46–55. 19333911 10.1002/hep.22951

[176] Lau GKK, Piratvisuth T, Luo KX, Marcellin P, Thongsawat S, Cooksley G, et al. Peginterferon alfa-2a, lamivudine, and the combination for HBeAg-positive chronic hepatitis B. N Engl J Med. 2005;352(26):2682–95. 15987917 10.1056/NEJMoa043470

[177] Mücke MM, Backus LI, Mücke VT, Coppola N, Preda CM, Yeh ML, et al. Hepatitis B virus reactivation during direct-acting antiviral therapy for hepatitis C: a systematic review and meta-analysis. Lancet Gastroenterol Hepatol. 2018;3(3):172–80. 29371017 10.1016/S2468-1253(18)30002-5

[178] Pockros PJ. Black box warning for possible HBV reactivation during DAA therapy for chronic HCV infection. Gastroenterol Hepatol. 2017;13(9):536–40. PMC5635429 29038644

[179] AASLD. Recommendations for Testing, Managing, and Treating Hepatitis C. 2023. p. 1–256.

[180] EASL 2017 Clinical Practice Guidelines on the management of hepatitis B virus infection. J Hepatol. 2017 Aug;67(2):370–98. 10.1016/j.jhep.2017.03.021 28427875

[181] Osmani Z, Boonstra A. Recent Insights into the Role of B Cells in Chronic Hepatitis B and C Infections. Pathog (Basel, Switzerland). 2023 Jun;12(6). 10.3390/pathogens12060815 PMC10303100 37375505

[182] Ito M, Kusunoki H, Mochida K, Yamaguchi K, Mizuochi T. HCV infection and B-cell lymphomagenesis. Adv Hematol. 2011;2011:835314. 21789042 10.1155/2011/835314 PMC3140784

[183] Agematsu K, Hokibara S, Nagumo H, Komiyama A. CD27: a memory B-cell marker. Immunol Today. 2000;21(5):204–6. 10782048 10.1016/s0167-5699(00)01605-4

[184] Fayed HL, Abd El Ghany SM, Nasser AM, El-gendy NA. Effectiveness of direct-acting antiviral (DAA) agents on hepatitis C virus-related mixed cryoglobulinemia syndrome: one-year follow-up. Egypt Rheumatol. 2023;45(2):139–43.

[185] Jensen SB, Serre SBN, Humes DG, Ramirez S, Li YP, Bukh J, et al. Substitutions at NS3 Residue 155, 156, or 168 of hepatitis C virus genotypes 2 to 6 induce complex patterns of protease inhibitor resistance. Antimicrob Agents Chemother. 2015;59(12):7426–36. 10.1128/aac.01953-15. 26392503 10.1128/AAC.01953-15 PMC4649233

[186] Jensen SB, Fahnøe U, Pham L V, Serre SBN, Tang Q, Ghanem L, et al. Evolutionary Pathways to Persistence of Highly Fit and Resistant Hepatitis C Virus Protease Inhibitor Escape Variants. Hepatology [Internet]. 2019;70(3). Available from: https://journals.lww.com/hep/fulltext/2019/09000/evolutionary_pathways_to_persistence_of_highly_fit.2.aspx 10.1002/hep.30647 PMC6772116 30964552

[187] Dietz J, Susser S, Vermehren J, Peiffer KH, Grammatikos G, Berger A, et al. Patterns of resistance-associated substitutions in patients with chronic HCV infection following treatment with direct-acting antivirals. Gastroenterology. 2018;154(4):976-988.e4. 29146520 10.1053/j.gastro.2017.11.007

[188] Soumana DI, Kurt Yilmaz N, Ali A, Prachanronarong KL, Schiffer CA. Molecular and dynamic mechanism underlying drug resistance in genotype 3 hepatitis C NS3/4A protease. J Am Chem Soc. 2016;138(36):11850–9. 10.1021/jacs.6b06454. 27512818 10.1021/jacs.6b06454 PMC5221612

[189] Izhari MA. Molecular mechanisms of resistance to direct-acting antiviral (DAA) drugs for the treatment of hepatitis C virus infections. Diagnostics [Internet]. 2023;13(19). Available from: https://www.mdpi.com/2075-4418/13/19/3102 10.3390/diagnostics13193102 PMC10572573 37835845

[190] Boonma T, Nutho B, Darai N, Rungrotmongkol T, Nunthaboot N. Exploring of paritaprevir and glecaprevir resistance due to A156T mutation of HCV NS3/4A protease: molecular dynamics simulation study. J Biomol Struct Dyn. 2022;40(12):5283–94. 10.1080/07391102.2020.1869587. 33430709 10.1080/07391102.2020.1869587

[191] Soni S, Singh D, Aggarwal R, Veerapu NS. Enhanced fitness of hepatitis C virus increases resistance to direct-acting antivirals. J Gen Virol. 2022;103(2):1699. 10.1099/jgv.0.001699 35133954

[192] Fernandez-Antunez C, Wang K, Fahnøe U, Mikkelsen LS, Gottwein JM, Bukh J, et al. Characterization of multi-DAA resistance using a novel hepatitis C virus genotype 3a infectious culture system. Hepatology [Internet]. 2023;78(2). Available from: https://journals.lww.com/hep/fulltext/2023/08000/characterization_of_multi_daa_resistance_using_a.24.aspx 10.1097/HEP.0000000000000353 36999539

[193] Pham LV, Pedersen MS, Fahnøe U, Fernandez-Antunez C, Humes D, Schønning K, et al. HCV genome-wide analysis for development of efficient culture systems and unravelling of antiviral resistance in genotype 4. Gut. 2022;71(3):627LP – 642. 33833066 10.1136/gutjnl-2020-323585 PMC8862099

[194] Kyuregyan KK, Kichatova VS, Karlsen AA, Isaeva OV, Solonin SA, Petkov S, et al. Factors influencing the prevalence of resistance-associated substitutions in NS5A protein in treatment-naive patients with chronic hepatitis C. Biomedicines. 2020;8(4):80. 32272736 10.3390/biomedicines8040080 PMC7235841

[195] Costa GL, Sautto GA. Exploring T-cell immunity to Hepatitis C virus: insights from different vaccine and antigen presentation strategies. Vaccines. 2024. 10.3390/vaccines12080890. 39204016 10.3390/vaccines12080890 PMC11359689

[196] Dai L, Du Y, Qi H, Huber CD, Wu NC, Wang E, et al. Adaptive potential of a drug-targeted viral protein as a function of environmental stress. bioRxiv [Internet]. 2018 Jan 1;78428. Available from: http://biorxiv.org/content/early/2018/01/09/078428.abstract

[197] Virzì A, Suarez AAR, Baumert TF, Lupberger J. Oncogenic signaling induced by HCV infection. Viruses. 2018;10(10):1–21. 10.3390/v10100538 PMC6212953 30279347

[198] Hlady RA, Zhao X, El Khoury LY, Luna A, Pham K, Wu Q, et al. Interferon drives HCV scarring of the epigenome and creates targetable vulnerabilities following viral clearance. Hepatology. 2022;75(4):983–96. 34387871 10.1002/hep.32111 PMC9416882

[199] Macek Jilkova Z, Saleem K, Afzal S, Decaens T. Predictive factors for hepatocellular carcinoma development after direct-acting antiviral treatment of HCV. Livers. 2021;1(4):313–21.

[200] Janjua NZ, Wong S, Darvishian M, Butt ZA, Yu A, Binka M, et al. The impact of SVR from direct-acting antiviral- and interferon-based treatments for HCV on hepatocellular carcinoma risk. J Viral Hepat. 2020;27(8):781–93. 32187430 10.1111/jvh.13295

[201] Hamdane N, Jühling F, Crouchet E, El Saghire H, Thumann C, Oudot MA, et al. HCV-induced epigenetic changes associated with liver cancer risk persist after sustained virologic response. Gastroenterology. 2019;156(8):2313-2329.e7. 30836093 10.1053/j.gastro.2019.02.038 PMC8756817

[202] Jühling F, Hamdane N, Crouchet E, Li S, El Saghire H, Mukherji A, et al. Targeting clinical epigenetic reprogramming for chemoprevention of metabolic and viral hepatocellular carcinoma. Gut. 2021;70(1):157–69. 32217639 10.1136/gutjnl-2019-318918 PMC7116473

[203] Perez S, Kaspi A, Domovitz T, Davidovich A, Lavi-Itzkovitz A, Meirson T, et al. Hepatitis C virus leaves an epigenetic signature post cure of infection by direct-acting antivirals. PLoS Genet. 2019;15(6):1–28. 10.1371/journal.pgen.1008181 PMC6602261 31216276

[204] Oltmanns C, Liu Z, Mischke J, Tauwaldt J, Mekonnen YA, Urbanek-Quaing M, et al. Reverse inflammaging: long-term effects of HCV cure on biological age. J Hepatol. 2023;78(1):90–8. 10.1016/j.jhep.2022.08.042. 36152762 10.1016/j.jhep.2022.08.042

[205] Haga H, Sato H, Koseki A, Saito T, Okumoto K, Hoshikawa K, et al. A machine learning-based treatment prediction model using whole genome variants of hepatitis C virus. PLoS One. 2020;15(11):e0242028. 10.1371/journal.pone.0242028. 33152046 10.1371/journal.pone.0242028 PMC7644079

[206] Lu MY, Huang CF, Hung CH, Tai C, Mo LR, Kuo HT, et al. Artificial intelligence predicts direct-acting antivirals failure among hepatitis C virus patients: a nationwide hepatitis C virus registry program. Clin Mol Hepatol. 2024;30(1):64–79. 10.3350/cmh.2023.0287. 38195113 10.3350/cmh.2023.0287 PMC10776298

[207] Adiwijaya BS, Kieffer TL, Henshaw J, Eisenhauer K, Kimko H, Alam JJ, et al. A viral dynamic model for treatment regimens with direct-acting antivirals for chronic hepatitis C infection. PLoS Comput Biol. 2012;8(1):e1002339. 10.1371/journal.pcbi.1002339. 22241977 10.1371/journal.pcbi.1002339 PMC3252270

[208] Ke R, Loverdo C, Qi H, Sun R, Lloyd-Smith JO. Rational design and adaptive management of combination therapies for hepatitis C virus infection. PLoS Comput Biol. 2015;11(6):e1004040. 10.1371/journal.pcbi.1004040. 26125950 10.1371/journal.pcbi.1004040 PMC4488346

[209] Kurosaki M, Tanaka Y, Nishida N, Sakamoto N, Enomoto N, Honda M, et al. Pre-treatment prediction of response to pegylated-interferon plus ribavirin for chronic hepatitis C using genetic polymorphism in IL28B and viral factors. J Hepatol. 2011;54(3):439–48. 21129805 10.1016/j.jhep.2010.07.037

[210] O’Brien TR, Everhart JE, Morgan TR, Lok AS, Chung RT, Shao Y, et al. An IL28B genotype-based clinical prediction model for treatment of chronic hepatitis C. PLoS One. 2011;6(7):e20904. 21760886 10.1371/journal.pone.0020904 PMC3132753

[211] Ochi H, Hayes CN, Abe H, Hayashida Y, Uchiyama T, Kamatani N, et al. Toward the establishment of a prediction system for the personalized treatment of chronic hepatitis C. J Infect Dis. 2012;205(2):204–10. 22124128 10.1093/infdis/jir726

[212] Ashraf MU, Salman HM, Khalid MF, Khan MHF, Anwar S, Afzal S, et al. CRISPR-Cas13a mediated targeting of hepatitis C virus internal-ribosomal entry site (IRES) as an effective antiviral strategy. Biomed Pharmacother. 2021;136:111239. 33454599 10.1016/j.biopha.2021.111239

[213] Sautto G, Tarr AW, Mancini N, Clementi M. Structural and antigenic definition of hepatitis C virus E2 glycoprotein epitopes targeted by monoclonal antibodies. Clin Dev Immunol. 2013;2013:450763. 10.1155/2013/450963 PMC3722892 23935648

[214] Sautto GA, Wisskirchen K, Clementi N, Castelli M, Diotti RA, Graf J, et al. Chimeric antigen receptor (CAR)-engineered t cells redirected against hepatitis C virus (HCV) E2 glycoprotein. Gut. 2016;65(3):512–23. 25661083 10.1136/gutjnl-2014-308316 PMC4789830

